# Applicability of Nanoemulsions for the Incorporation of Bioactive Compounds in Cosmetics: A Review

**DOI:** 10.1021/acsomega.5c08224

**Published:** 2026-01-20

**Authors:** Aniely Cristina de Souza, Caroline Casagrande Sipoli, Ana Caroline Raimundini Aranha, Rafael Block Samulewski, Gustavo Nogueira da Silva, Rafael Oliveira Defendi, Maria Carolina Sérgi Gomes, Rúbia Michele Suzuki

**Affiliations:** † Postgraduate Program in Chemical Engineering (PPGEQ-AP), Federal Technological University of Paraná (UTFPR), Rua Marcílio Dias, 635, CEP 86812-460 Apucarana, PR, Brazil; ‡ Postgraduate Program in Chemical Engineering, Department of Chemical Engineering, State University of Maringá, Av. Colombo 5790, Bloco D90, CEP 87020-900 Maringá, PR, Brazil

## Abstract

Plants are important sources of metabolites used in the cosmetics, food, and pharmaceutical industries, especially in cosmetics, where bioactive compounds offer benefits for the skin, such as protection against environmental stresses. The term “cosmeceutical” has emerged to describe products that combine aesthetic effects and dermatological treatments. With the growth of the cosmetics industry, the demand for ingredients that combat the signs of aging and oxidative stress – the main cause of skin aging – has increased. Bioactive compounds, such as phenols, flavonoids, and carotenoids, have antioxidant properties that are widely used to control the skin’s aging process, triggered by environmental factors or the body’s own metabolism, leading to excessive production of free radicals and, consequently, oxidative stress. However, incorporating these lipophilic compounds into water-based cosmetic formulas presents major challenges, including poor solubility, low stability, limited skin penetration, and rapid degradation. Nanoemulsions overcome these limitations by enabling droplet sizes of 20–200 nm through high-energy (e.g., high-pressure homogenization, ultrasonication) or low-energy (e.g., phase inversion temperature, spontaneous emulsification) methods. Their significance in cosmeceuticals lies in enhanced skin penetration, improved bioavailability of lipophilic actives, and prolonged product stability, making them ideal for antiaging creams, sunscreens, and moisturizers. This review article aims to address antioxidant compounds, their cosmetic applications, and the techniques used to obtain them, including characterization methods, validation of nanoemulsions, the main difficulties, and future prospects.

## Graphical abstract

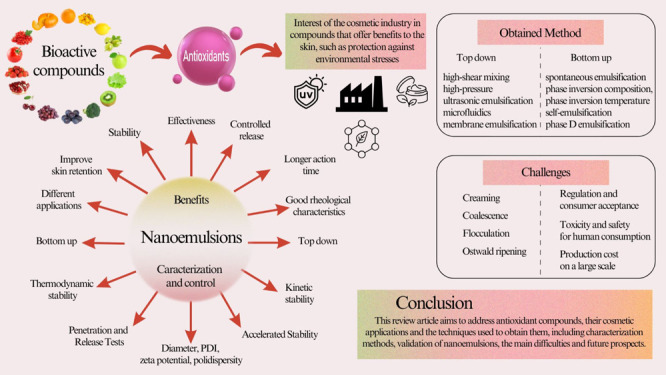

## 1 Introduction

Plants are widely recognized as major sources of primary and secondary metabolites used as active ingredients in the cosmetic, food, and pharmaceutical industries. The cosmetic industry, in particular, has shown a growing interest in these compounds because the same agents that protect plants against environmental stresses can offer similar benefits to human skin.^1^ This has led to the emergence of the term ″cosmeceutical,″ which refers to products formulated to provide aesthetic effects like cosmetics and treat dermatological conditions like pharmaceuticals.^2^

The use of bioactive compounds to improve skin appearance dates back 6,000 years. Currently, the increasing concern for beauty has driven rapid growth in the global cosmetic industry, with a forecasted value of 716 billion dollars by 2025.^3^ This growth aligns with the rising demand from consumers for ingredients that not only protect the skin but also improve the effects of aging.

The aging process is mainly the result of the accumulation of oxidative stress,^4^ which is one of the major dermatological concerns. It is characterized by the appearance of fine wrinkles, loss of skin elasticity and tone, and age spots. This process can be triggered by internal factors such as time, hormones, and genetics, or external factors such as exposure to radiation and pollution.^5,6^

Oxidative stress is characterized by the accumulation of Reactive Oxygen Species (ROS) beyond the capacity of a biological system to neutralize them. Generally, 1.5% to 5% of the oxygen consumed by cells is converted into ROS.^4^ These species are produced from metabolism as signaling molecules (cellular messengers). However, when generated in higher concentrations due to exposure to environmental stress factors,^7^ they can damage macromolecules, such as oxidizing proteins, nucleic acids, and lipids. This damage can lead to skin aging and various diseases, including rheumatoid arthritis, atherosclerosis, neurodegenerative diseases, cancer, obesity, and type 2 diabetes mellitus.^8^

To combat the excess reactive oxygen species (ROS), the body utilizes compounds that can interfere with the oxidation process, preventing or removing oxidative damage either endogenously or exogenously.^9^ Plant bioactive compounds are well-known for their antioxidant properties, derived from secondary metabolites—chemical substances not directly involved in the plant growth process but produced in response to environmental conditions. The most well-known plant-derived antioxidants (phyto-antioxidants) are phenolic compounds (flavonoids and nonflavonoids), terpenoid groups (with carotenoids being the most recognized), and vitamins (A, E, and C).^10,11^

Bioactive compounds, including phenolic acids, flavonoids, carotenoids, and vitamins, have garnered attention in cosmetics due to their beneficial effects on skin health. Phenolic compounds are particularly recognized for their powerful antioxidant capacity, helping to protect cells from oxidative stress—a significant factor in skin aging and cellular damage. These compounds act by neutralizing ROS, reducing inflammation, and supporting skin integrity, which makes them suitable for antiaging formulations.^11,12^

Flavonoids, a prominent subgroup of polyphenols, are widely used for their antioxidant and anti-inflammatory properties. These compounds, found abundantly in fruits and vegetables, are known to stabilize free radicals and support collagen synthesis, contributing to skin elasticity and reduced signs of aging. For instance, quercetin and catechins (Figure 1) provide antioxidative protection, making them valuable for skin rejuvenation and protection in cosmetic applications.^13^

**Figure 1:**
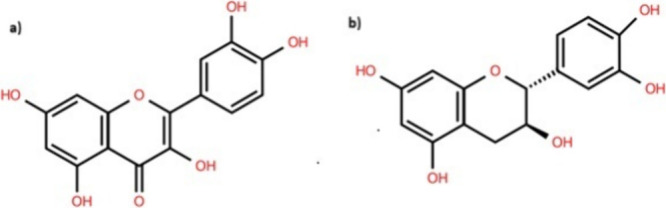
Molecular structures. (a) Quercetin; (b) catechin.

Carotenoids and vitamins complement the effects of phenolics in skincare. Carotenoids like beta-carotene provide UV protection and help in improving skin tone by reducing pigmentation. Meanwhile, vitamins, especially A, C, and E, are essential for collagen production, cellular repair, and moisture retention, with Vitamin C widely used for its brightening effects and environmental protection qualities. The inclusion of these compounds in skincare can enhance skin health and counteract signs of aging.^13,14^ Representative carotenoids and vitamins are shown in Figure 2.

**Figure 2:**
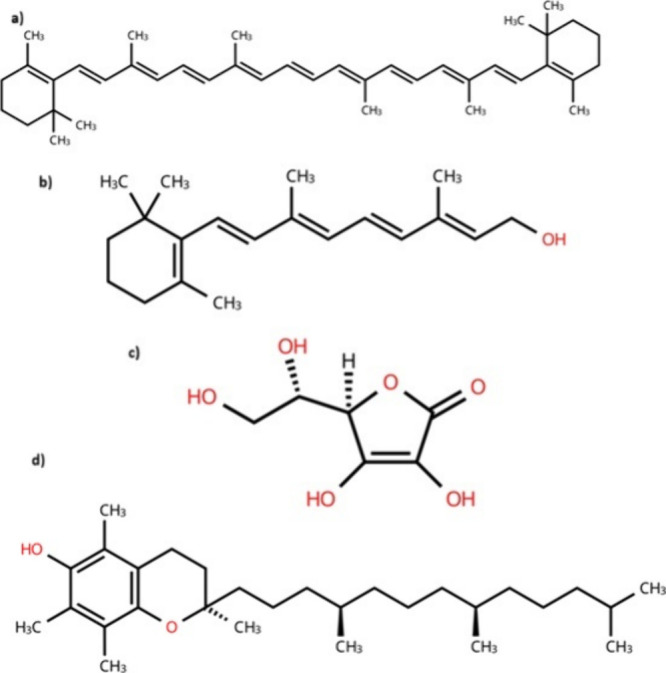
Molecular structures. (a) Beta-carotene; (b) vitamin A; (c) vitamin C; (d) vitamin E.

Despite their wide range of applications, one of the frequent challenges in cosmetics, especially with liposoluble actives, is the difficulty of incorporating them into water-based cosmetics, limiting their use. Consequently, various techniques, such as nanotechnology, have been employed to overcome these limitations.^15^

Nanotechnology refers to materials in the nanoscale range.^16^ Different techniques can be applied in developing nanoparticulate systems, among which nanoemulsions stand out for extending product shelf life due to their small droplet size. These systems, characterized by a mixture of water, oil, and surfactant, have shown promising results for developing various pharmaceuticals and in biotechnology, as well as in cosmetics, including makeup, cleansers, sunscreens, and moisturizers.^17^

The use of nanotechnology as an alternative to incorporate actives with limiting usage characteristics, such as vegetable oils and lipophilic vitamins, into systems with good rheological and sensory properties like nanoemulsions, allows the development of formulations capable of maximizing the benefits of these compounds and opening new administration routes. In light of the above, the general objective of this review article is to discuss antioxidant compounds, from synthetic to natural, their cosmetic applications, evaluate nanotechnology, nanoparticulate systems, nanoemulsions, and techniques for characterizing these nanoemulsions and validation methods.

## 2 Nanoparticle Systems and Their Designs

### 2.1 Nanotechnology and Nanoparticle Systems

Nanoparticle systems are engineered structures typically below 100 nm, designed to enhance the delivery, stability, and performance of bioactive compounds in cosmetics, pharmaceuticals, and biotechnology. Their morphology, composition, and surface functionality enable controlled release and targeted delivery. Among the most relevant systems are solid lipid nanoparticles, polymeric nanoparticles, nanoliposomes, nanostructured lipid carriers, and particularly nanoemulsions, which stand out for their kinetic stability, ease of preparation, and excellent performance in cosmetic formulations.^18–32^

#### 2.1.1 Types of Nanoparticle Systems

Nanoparticulate systems can be classified according to their characteristics and composition (Table 1). Among the most commonly used organic systems for the development of cosmetics are solid lipid nanoparticles, polymeric nanoparticles, nanoliposomes, nanostructured lipid carriers, and nanoemulsions.^33–35^ A schematic comparison of the main nanoparticle systems is presented in Figure 3.

**Figure 3:**
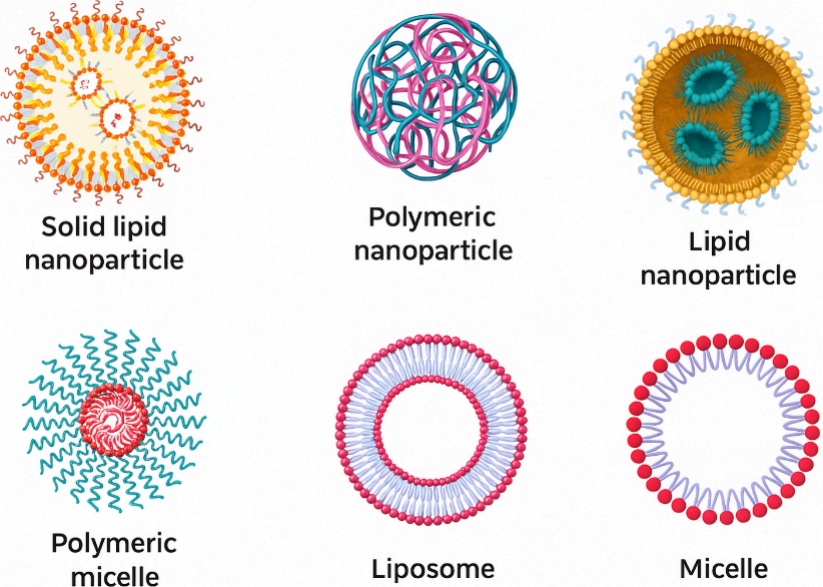
Types of nanoparticle systems.

**Table 1:** Comparative Overview of the Main Nanoparticle Systems Used in Cosmetics

System	Composition/Structure	Advantages	Limitations	Typical Applications in Cosmetics
**Solid Lipid Nanoparticles (SLNs)**	Solid lipid core stabilized by surfactants	High biocompatibility; controlled release; good protection of actives	Possible drug expulsion during storage; limited loading capacity	Antiaging creams, sunscreens
**Nanostructured Lipid Carriers (NLCs)**	Blend of solid and liquid lipids	Improved loading capacity; better physical stability than SLNs	Complex formulation; potential for polymorphic transitions	Moisturizers, antioxidant formulations
**Polymeric Nanoparticles**	Biodegradable or synthetic polymers (PLGA, chitosan, etc.)	Controlled release; tunable surface properties	Possible cytotoxicity of residual monomers or solvents	Antiacne treatments, skin repair
**Nanoliposomes**	Phospholipid bilayers encapsulating aqueous/lipid phases	High biocompatibility; encapsulation of hydrophilic and lipophilic actives	Low physical stability; oxidation of lipids	Delivery of vitamins and peptides
**Nanoemulsions**	Oil and water phases stabilized by surfactants (20–200 nm)	Easy preparation; high kinetic stability; enhanced skin penetration; transparent appearance; improved bioavailability	Thermodynamically unstable; sensitive to surfactant type and concentration	Serums, sunscreens, moisturizers, antiaging products

Solid lipid nanoparticles are colloidal systems composed of biodegradable solid lipids, surfactants, and cosurfactants as stabilizers. Their size can vary between 5 and 100 nm, and they can incorporate both hydrophilic and lipophilic actives.^35^ They are promising as nanovaccine delivery systems,^36^ as well as for applications in the treatment of skin cancer and hyperpigmentation disorders.^37,38^ Techniques used for their production include high-pressure homogenization combined with ultrasound, solvent evaporation, and microfluidics.^38–41^

Polymeric nanoparticles have the potential for various applications, whether for diagnosis or drug delivery. The average size can range from 100 to 300 nm, but sizes smaller than 50 nm can also be obtained.^42^ Among the advantages, controlled release, targeting the active ingredient to the site of action, and the possibility of combining different drugs can be highlighted.^43^

The development of polymeric nanoparticles can ensure small particle sizes that facilitate entry into the cellular environment, and depending on the technique used, nanospheres—systems where active ingredients and polymers are uniformly dispersed, or nanocapsules—systems where a polymer shell surrounds the actives can be obtained.^44,45^ It is important to note that drug release depends on the degradation rate of the polymer used. In this case, biodegradable and biocompatible polymers are preferred as they ensure complete release from the organism.^43,45^

Generally, the production of this system can occur through nanoprecipitation, solvent diffusion, emulsification/reverse salting-out, solvent evaporation, and commonly requires a preformed polymer and organic solvents in the preparation.^42^

Nanoliposomes are widely used for drug delivery. They are circular systems with an aqueous core, composed of one or more lipid bilayers. When composed of amphiphilic lipids, they form spontaneously, similar to biological membranes, promoting greater biocompatibility.^46,47^ These systems are capable of encapsulating hydrophilic and lipophilic drugs, either in the core or between the lipid bilayers.^48^ The particle size can range from 1 to 100 nm,^49^ and among the most commonly used lipids in the formation of nanoliposomes are phosphatidylethanolamine, phosphatidylserine, phosphatidylglycerol, and phosphatidylcholine. Their spontaneous formation and low toxicity make them good candidates for the encapsulation of active compounds.^48^

Nanostructured lipid carriers are the second generation of solid lipid nanoparticles.^50^ They are obtained from mixtures of solid lipids, liquid lipids, and surfactants,^50,51^ allowing for a higher load of active compounds and a lower amount of water. They are capable of forming three types of systems: (a) imperfect crystal, where a completely disordered structure is formed, (b) amorphous type, where certain lipids create a noncrystalline structure that prevents the expulsion of the active compound, and (c) multiple type, when the solubility of the active compounds is higher in the liquid lipid than in the solid lipid.^51^ Among the advantages, they offer better physical stability, controlled particle size, elimination of organic solvents, compatibility with hydrophilic and lipophilic actives, and easy preparation.^52^

## 3 Nanoemulsions: Definition, Structure, and Characteristics

Nanoemulsions are colloidal dispersions consisting of two immiscible liquids—typically oil and water—stabilized by one or more surfactants to form droplets with diameters usually between 20 and 200 nm. Unlike conventional emulsions, nanoemulsions are thermodynamically unstable but kinetically stable systems, meaning that they resist separation over long periods due to their extremely small droplet size and high surface area-to-volume ratio. The nanometric scale provides optical transparency or translucency and enhances the delivery of lipophilic and hydrophilic active compounds.^53–55^

Structurally, a nanoemulsion comprises a dispersed phase (oil or water) finely distributed within a continuous phase, with the surfactant molecules positioned at the interface to lower interfacial tension and prevent droplet coalescence. Depending on the composition, nanoemulsions can exist as oil-in-water (O/W), water-in-oil (W/O), or multiple (W/O/W, O/W/O) systems (Figure 4).^53–55^

**Figure 4:**
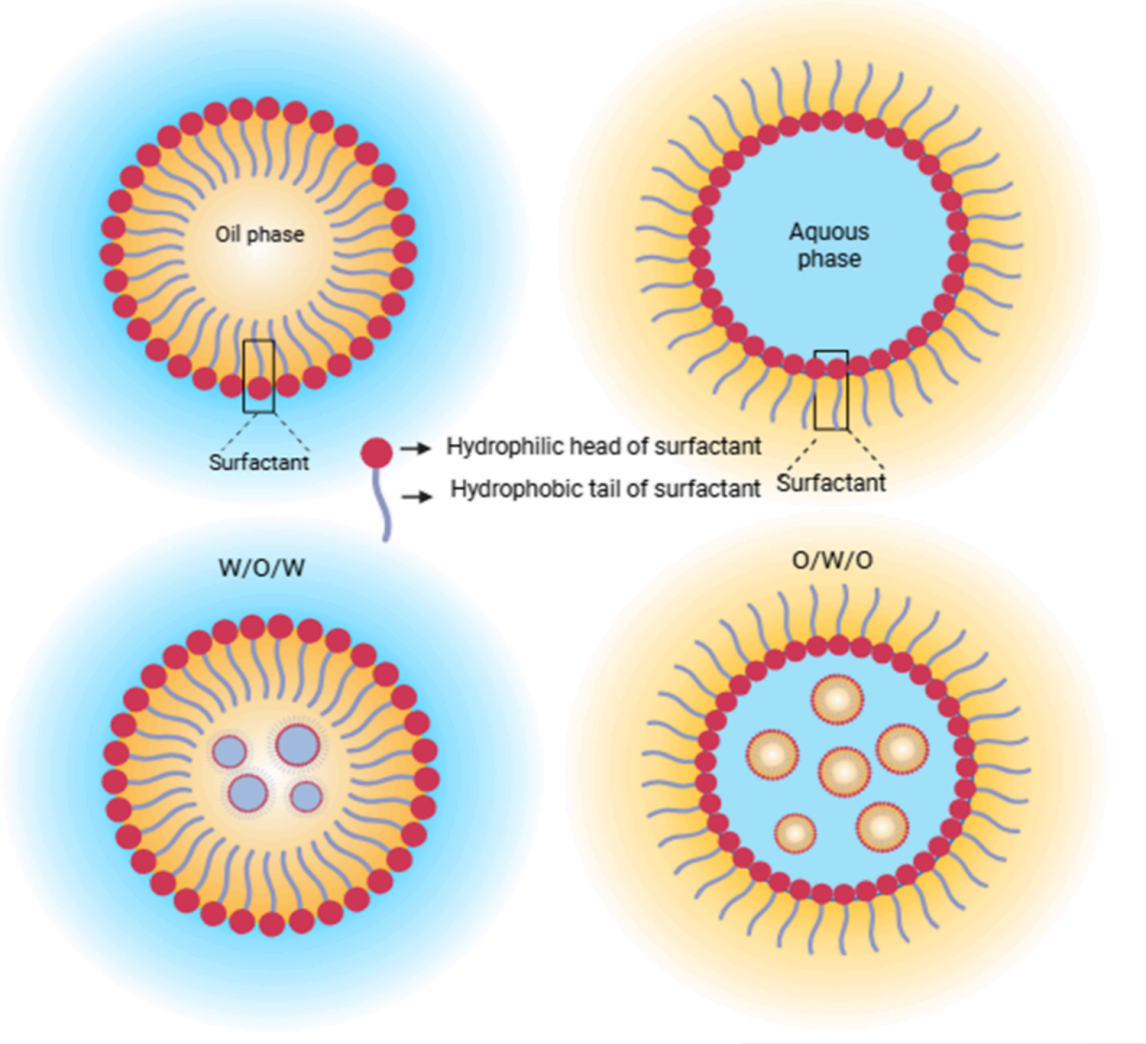
Types of nanoemulsions.

Their physicochemical features—small droplet size, large interfacial area, and tunable rheology—confer advantages such as improved solubility of poorly water-soluble ingredients, enhanced penetration through the skin barrier, controlled release, and extended product stability. For these reasons, nanoemulsions are widely used in cosmetics for the incorporation of antioxidants, vitamins, and UV-protective agents in formulations like serums, creams, and sunscreens.^53–57^

### 3.1 Emulsifiers

Emulsifiers are responsible for reducing the interfacial tension between the two phases, facilitating the formation of droplets and forming a coating around them to improve stability. They are generally amphiphilic molecules composed of a polar part, which projects into the aqueous phase, and an apolar tail that projects into the oil phase.^54^ The use of emulsifiers is essential for the formation of nanoemulsions, as they play a fundamental role in formation and stability, interfering with size, viscosity and electrical repulsion (Figure 5).^58^ They can be classified by molecular mass, chemical structure, mechanism of action and are subdivided into synthetic, natural, finely dispersed solids and auxiliary agents based on their chemical structure.^59^

**Figure 5:**
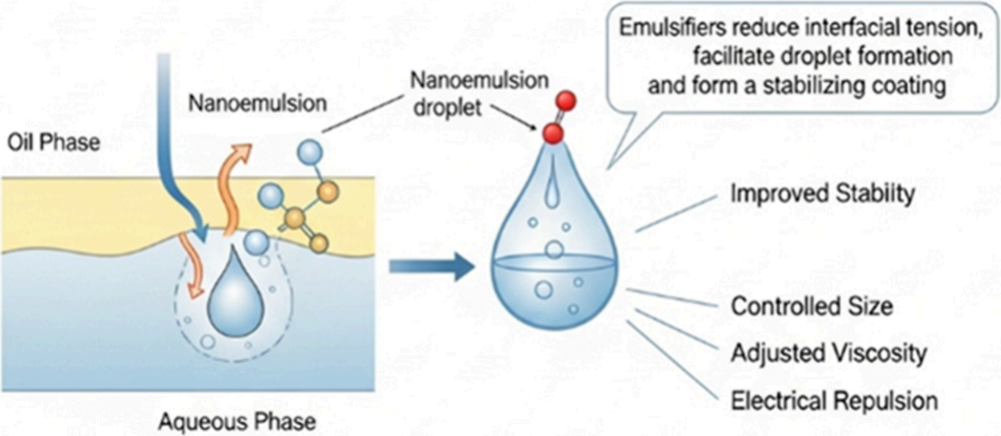
Emulsifier mechanism of action.

Among the most common natural emulsifiers include proteins, phospholipids, polysaccharides, lipopolysaccharides and bioemulsifiers such as saponins and rhamnolipids, which can be obtained from plants or produced by fermentation. Colloidal particles such as chitin, cellulose, starch and plant proteins also act as emulsifiers in oil-in-water emulsions, forming bonds that reduce coalescence and allow emulsions to form.^60^

Some hydrocolloid emulsifiers are obtained from vegetable sources, such as acacia, agar, pectin, carrageenan and lecithin, and others are of animal origin, such as lanolin and cholesterol, while there are those that are semisynthetic, such as methylcellulose and carboxymethylcellulose.^58,61^ Modified starch, pectin, gum arabic and galactomannans are natural hydrocolloids with emulsifying characteristics. Some emulsifiers are based on polysaccharides, such as gum arabic, beet pectin, corn fiber gum, soy polysaccharide, modified starch and nonionic methylcellulose. The high surface charges of these amphiphilic polysaccharides result from their molecular mass and dimensions, forming thick, hydrophilic biopolymers. Other emulsifiers are derived from proteins, including gelatin, casein, whey protein concentrates and isolates, beta-casein, sodium caseinate and calcium caseinate. The ratio of polar to nonpolar amino acids on the surface (surface hydrophobicity) can influence the surface activity of these protein biopolymers.^58,62,63^

Among the most frequently used emulsifiers are lecithins, which are natural compounds obtained from soybean seeds, eggs, milk, rapeseed, canola seed, sunflower, and cottonseed.^54,64^ They are amphiphilic phospholipids composed of two tails formed by fatty acids, which are lipophilic, linked to a glycerol backbone and a zwitterionic phosphate group, which is hydrophilic.^54^ In systems stabilized by lecithins, droplet aggregation is prevented due to electrostatic repulsion derived from the charge present in the phospholipid headgroup. This makes it stable at high temperatures and maintains its neutral pH; however, in acidic systems, it presents instability due to electrostatic repulsion and changes in surface charge.^65,66^ They can also be less effective when used alone, considering the intermediate HLB.^54^

The main phospholipid components of plant phospholipids are zwitterionic phosphatidylcholine, phosphatidylethanolamine, and anionic phosphatidylinositol, which also have a high degree of unsaturation. Sphingomyelin is an animal phospholipid that has recently gained prominence in liposome production.^67^

Synthetic emulsifiers can be classified into four categories: anionic, cationic, nonionic and amphoteric (Figure 6). Anionic emulsifiers have a negative charge on their hydrophilic part and include groups such as carboxylates, sulfonates, sulfates and phosphates. Cationic emulsifiers are positively charged and have preservative and antibacterial properties. Amphoteric emulsifiers have both positive and negative charges and can change from cationic to anionic or nonionic depending on the pH of the medium. Nonionic emulsifiers have no electrical charge and are made up of lipophilic and hydrophilic molecules.^68,69^ The small molecule surfactants have relatively low molecular weight. They also possess amphiphilic characteristics, meaning they consist of a polar headgroup and an apolar tail group. Due to their small size, they easily adsorb at oil–water interfaces during the homogenization process.^54^ Tweens and Spans are nontoxic at the necessary concentrations, making them widely used in food, pharmaceutical, and cosmetic formulations.^70^

**Figure 6:**
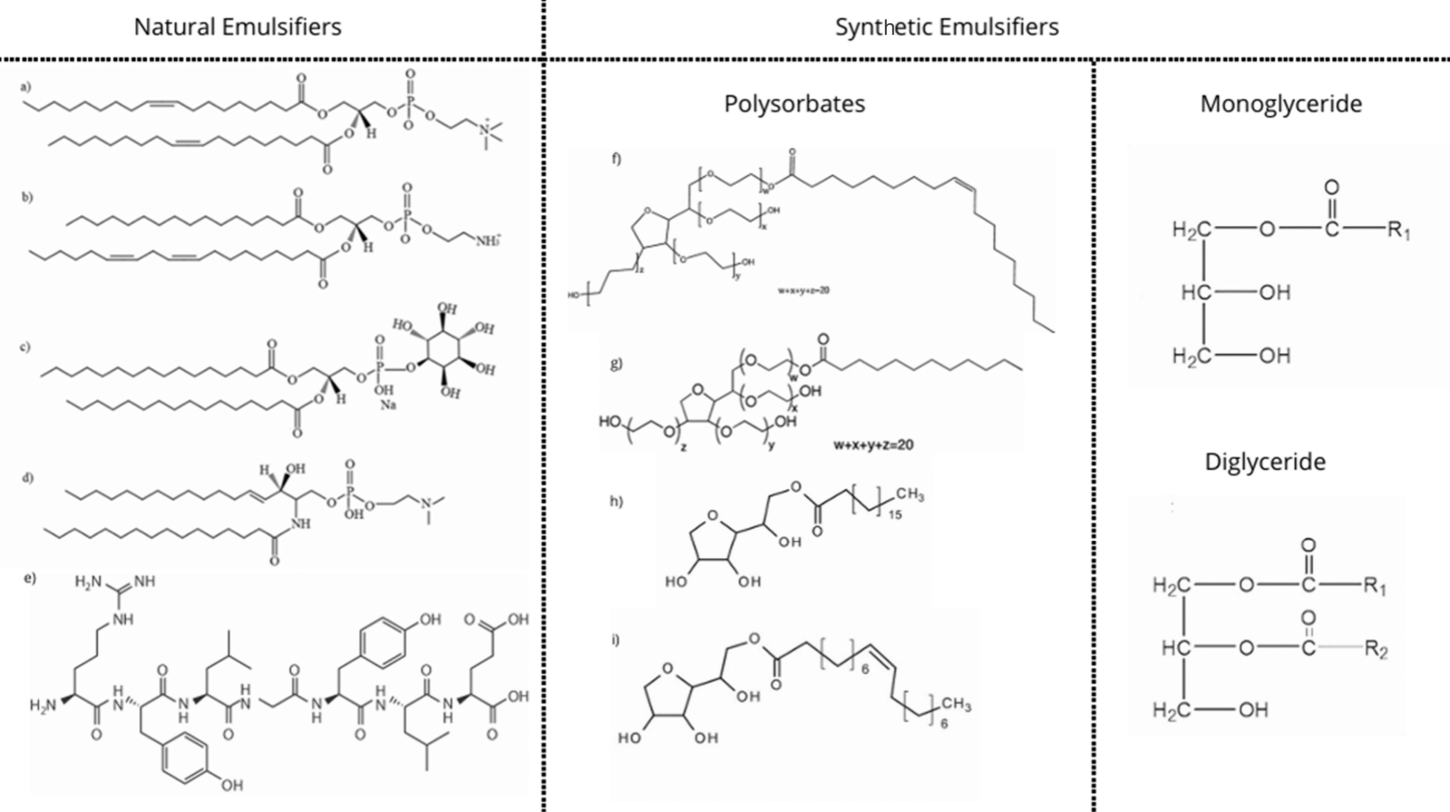
Classification of most common natural and synthetic emulsifiers: (a) phosphatidylcholines, (b) phosphatidylethanolamine, (c) anionic phosphatidylinositol, (d) animal phospholipid sphingomyelin, (e) casein, (f) Tween 20, (g) Tween 80, (h) Span 60, and (i) Span 80.

Finely dispersed solids, such as bentonite and magnesium hydroxide, are widely used in the formation of O/W emulsions, as they increase the viscosity of the dispersed phase and reduce the interaction between the phases, forming a layer of particles around the dispersed phase. Auxiliary agents, including fatty acids (such as stearic acid), fatty alcohols (such as cetyl alcohol) and esters (such as glyceryl monostearate), have limited emulsifying properties and must therefore be combined with more effective emulsifiers to ensure emulsion stability (Figure 7).^71^ The choice of emulsifiers and the determination of the concentration for the formulation directly affect the outcome of nanoemulsions, as does the method employed, which can be high or low energy. When these factors are correctly combined, desirable size and stability nanoemulsions are obtained.

**Figure 7:**
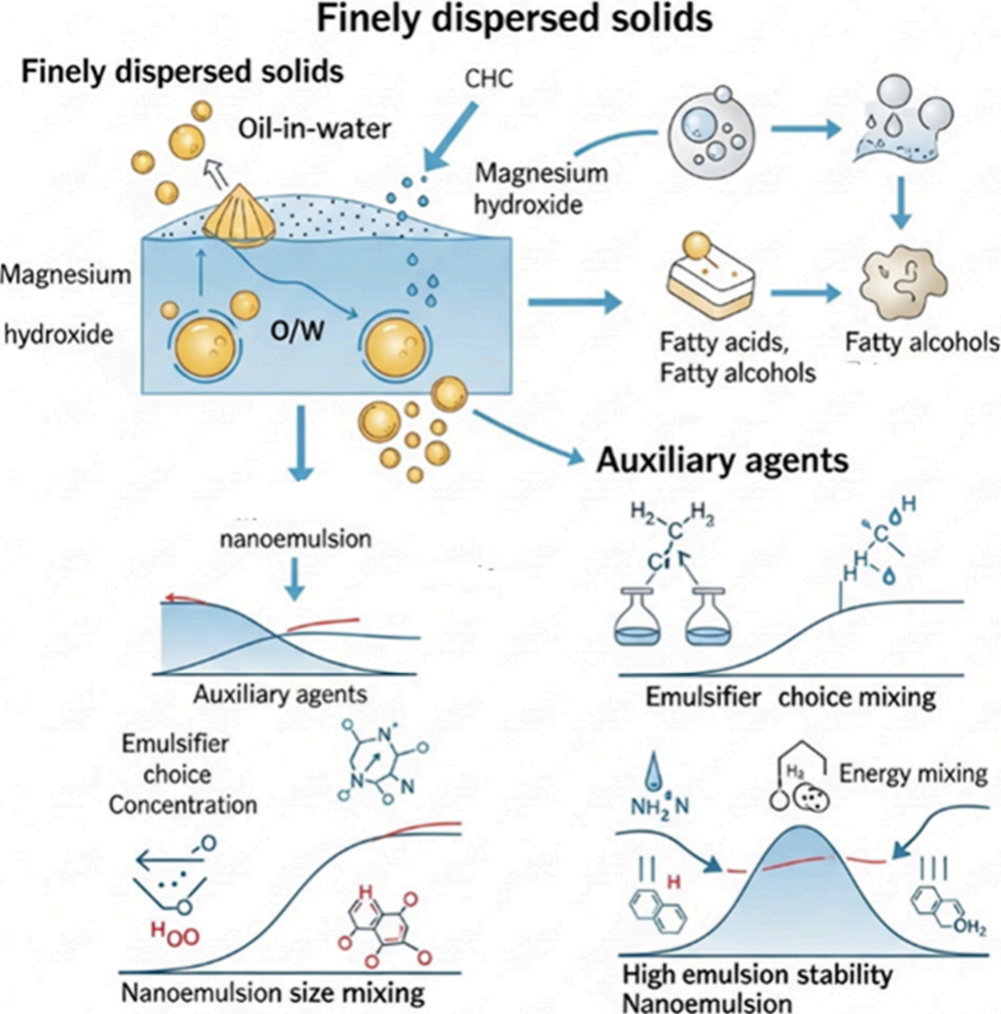
Roles of finely dispersed solids and auxiliary agents in nanoemulsion formulation.

### 3.2 Techniques for Obtaining Nanoemulsions

The preparation of nanoemulsions can be achieved using high-energy and low-energy methods (Figure 8). The high-energy method requires the use of equipment with significant disruptive forces, while low-energy methods alter the physicochemical properties of the system. Among the advantages, high-energy methods promote efficient formation of nanoemulsions more easily and quickly, whereas low-energy methods facilitate the nanoemulsification of temperature-sensitive systems without the need for specific equipment.^72,73^

**Figure 8:**
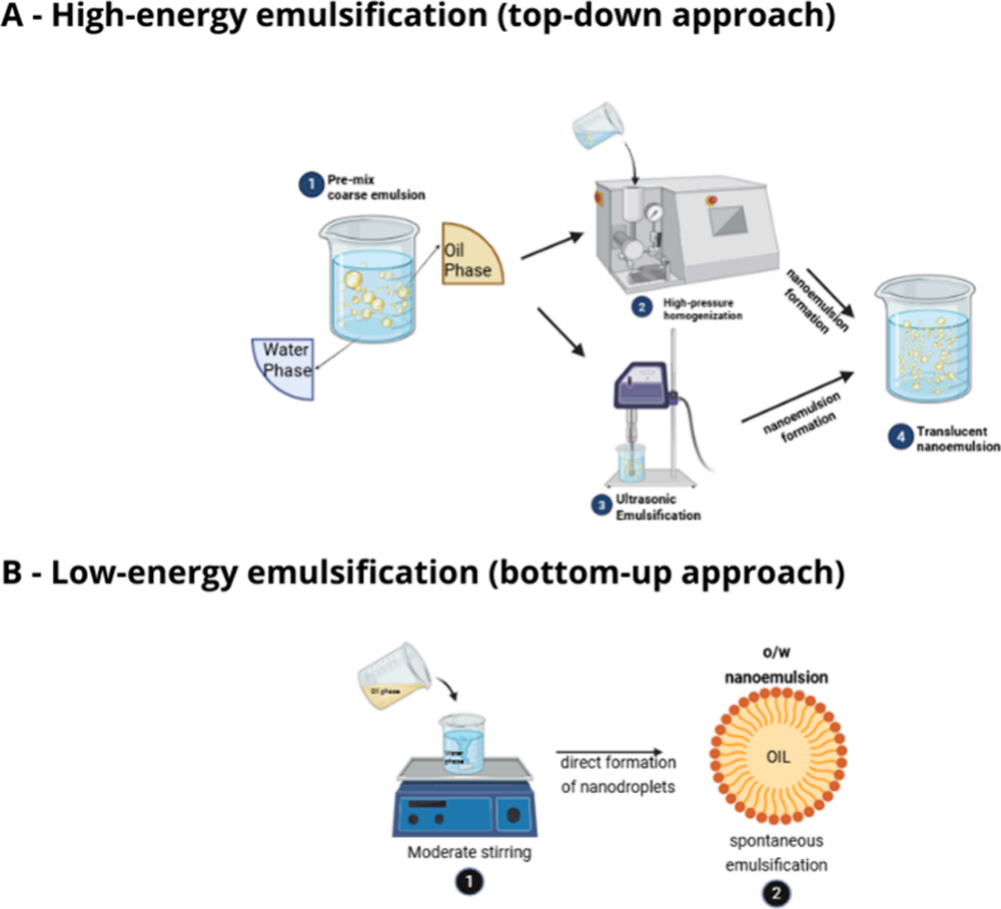
Nanoemulsion preparation: High-energy (a) vs low-energy methods (b).

For the development of a nanoemulsion using the high-energy method, it is necessary to use an aqueous phase, an oil phase, a surfactant, and the application of energy. The mechanical energy provided can create nanoemulsions with high kinetic energy, reducing the droplets to nanometric size. The techniques used include high-shear mixing, high-pressure homogenization, ultrasonic emulsification, microfluidics, and membrane emulsification. When the surfactant used is insufficient, the droplet sizes generally exceed the nanometric scale, leading to an instability phenomenon called coalescence. Despite this, through this technique, stability, particle size, rheology, and color can be controlled more effectively, in addition to reducing the risk of compound deterioration and inactivation.^19,72,74^

The commonly selected oils are those with high molecular weight and viscosity to facilitate the choice of surfactant. It is important to note that high-energy techniques are not recommended for the administration of heat-sensitive actives (especially drugs).^73^

Low-energy methods are most commonly applied in the formation of solid lipid nanoparticles and can be classified as isothermal and thermal techniques. The isothermal technique is suitable for thermally sensitive compounds. It is believed that the phenomenon responsible for formation is the chemical energy released during emulsification, causing the spontaneous curvature of surfactant molecules.^75^ This method requires low energy consumption, as it requires gentle stirring (around 1600 rpm). This technique includes spontaneous emulsification processes, phase inversion composition, phase inversion temperature, self-emulsification, and phase D emulsification.^72,76^

For spontaneous emulsification, the organic (or oil) phase and the surfactant are generally added to the aqueous phase, so that the rapid migration of miscible compounds to the aqueous phase increases the interfacial area of the phases, forming droplets.^77,78^ Solvents can be used during this process, in the presence or absence of surfactants (ouzo effect), and the order of mixing does not show a relevant effect on the process.^73^

Phase inversion composition can be described as an extension of spontaneous emulsification, as it is possible to obtain emulsions at room temperature without high-energy equipment and solvents. The system is assembled so that the mixture of oil and surfactant is under magnetic stirring at room temperature while water is added dropwise, initially forming W/O nanoemulsions followed by O/W, as the inversion point is reached with the increase in the amount of water (Figure 9). The nanoemulsion is formed due to interfacial tension, surfactant concentration and structure, and apparent viscosity.^73^

**Figure 9:**
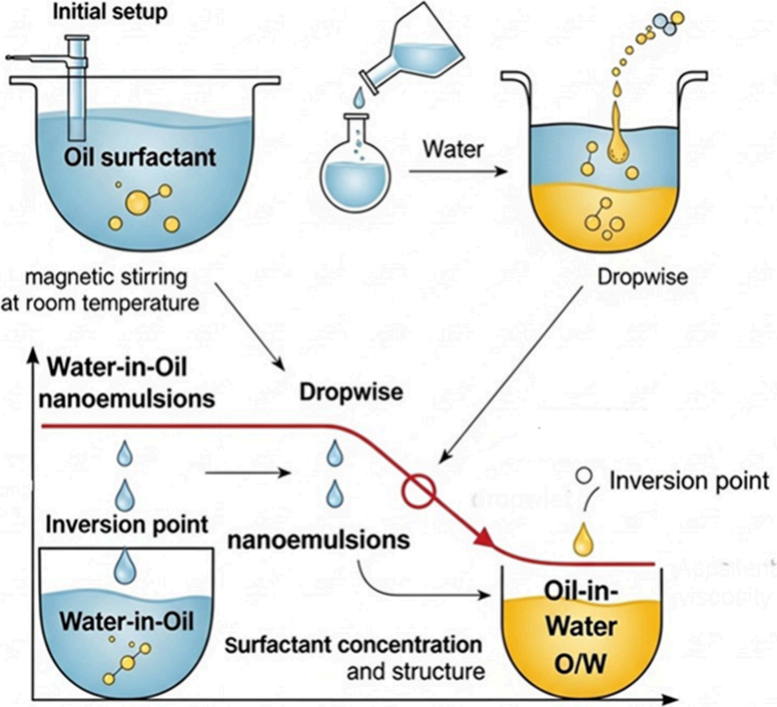
Phase inversion composition method.

The phase transition temperature is a system used to form O/W nanoemulsions. The aqueous phase, oil phase, and surfactants are stirred and gradually heated until they reach the phase inversion temperature (usually between 20 and 65 °C), then the mixture is rapidly cooled in an ice bath, forming the nanoemulsion.^72,73^ A disadvantage of this technique is that the system is sensitive to temperatures close to the inversion temperature, so the use of cosurfactants or nonionic surfactants is necessary, as the molecular geometry changes with temperature.^73^ This method shows low polydispersity indices and droplet size when compared to the previous technique.^77^

Self-emulsification is a technique that does not require high energy consumption and depends on the combination and concentration of the chosen lipid and surfactant (Figure 10). Also called microemulsion dilution, it consists of dilution at a constant temperature, where the O/W microemulsion is rapidly diluted with a large amount of water, decreasing the concentration of surfactant that maintains stability.^73,79^

**Figure 10:**
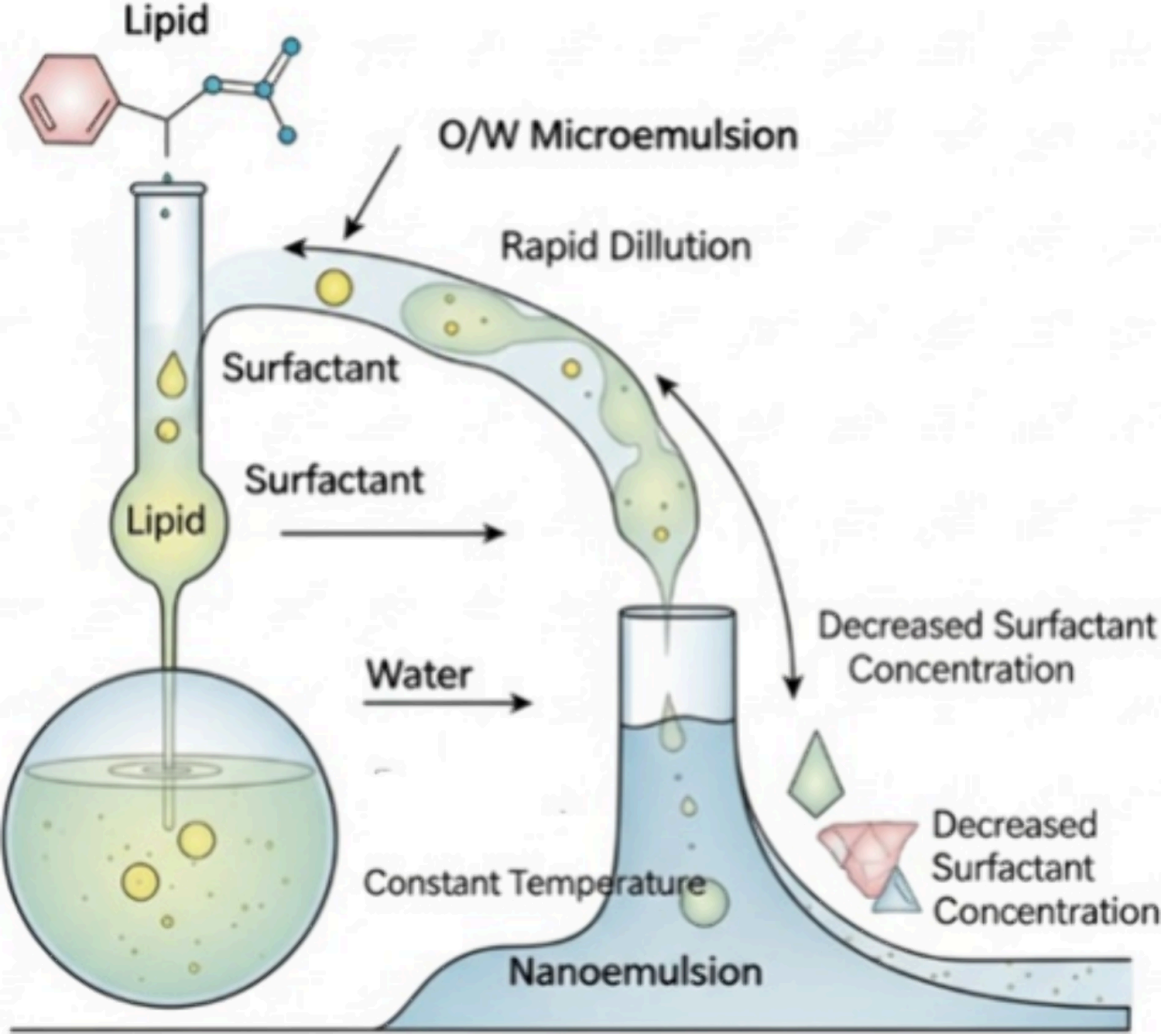
Self-emulsification.

Phase D emulsification was first reported in 1983.^73^ The system consists of the same base as the others: surfactant, water, and oil, but with the addition of an alkyl polyol as an extra for nanoemulsion formation. The technique does not require a solvent or high energy consumption, and it also requires a lower concentration of surfactant compared to other techniques.^80^

As described in Table 2, it is possible to verify updates regarding the use of the nanoemulsion system directed to the cosmetic area from different techniques and actives.

**Table 2:** Recent Applications of Nanoemulsions in the Cosmetic Field

System	Technique	Surfactants	Actives	Reference
Nanoemulsion incorporated in hydrogel	High-energy method using a high-shear homogenizer (Ultra-Turrax at 10000 rpm)	Phospholipid PL 80 and medium-chain triglycerides.	Coenzyme Q_10_	Dragicevic et al.^81^
Nanoemulsion	High-energy method (ultrasonic emulsification)	Sorbitan trioleate and polyoxyethylene oley ether	Curcumin	Chiu et al.^82^
Nanoemulsion	Low-energy method (phase D emulsification)	Tween 80 e and glycerin	Moringa seed oil	Inmuangkham et al.^83^
Nanoemulsion	Low-energy method (spontaneous emulsification)	(a)Transcutol and Tween 80, (b)Tween 80 and Span 80,(c)Transcutol and Labrasol	Rhodiola rosea extract	Iskandar et al.^16^
Nanoemulsion	High-energy method (ultrasonic emulsification)	Propylene glycol and PEG-40	*Passiflora quadrangularis* fruit extracts	Yanasan et al.^84^
Nanoemulsion	High-energy method using a high-shear homogenizer (Ultra-Turrax at 6000 rpm)	Tween 80 and Span 80	*M. pruriens* var. seed extracts	Chookiat et al.^85^

## 4 Characterization of Nanoemulsions

Based on the possible instabilities that can affect systems, directly interfering with the performance of the nanoemulsion, characterization techniques can be applied to monitor potential instabilities and determine the viability of the nanoemulsion for proper application. In the case of nanoemulsions for cosmetic application, tests determined by the National Health Surveillance Agency (ANVISA) are essential to ensure not only physical stability but also application safety.

### 4.1 Hydrodynamic Diameter

The diameter of nanoemulsions can vary depending on the system composition or technique used. In general, increasing pressure or rotation can significantly reduce the size; however, for certain emulsifiers (biopolymers), long periods of emulsification and very high pressures can hinder formation.^66^ Characteristics such as appearance and texture are directly related to droplet size. Authors indicate that nanoemulsions with a droplet size below 200 nm exhibit greater kinetic stability, prolonging shelf life, although long-term storage can cause instability phenomena.^86^

### 4.2 Zeta Potential

Zeta potential is a measure of electrostatic stability. The higher the surface charge of the nanoemulsion or material analyzed, the more stable the system. The zeta potential value is presented in modulus, but the charge results can be positive or negative, depending on the material.^87^ Generally, an appropriate zeta potential value should be around ± 30 mV, indicating that the system is stable,^66,88^ as high surface potential values on the droplets indicate strong repulsion between them, thus preventing flocculation and coalescence phenomena.^89^

### 4.3 Polydispersity

Polydispersity is a measure of uniformity given by the ratio between the standard deviation and the average size of the nanoemulsions; thus, the lower the polydispersity of a system, the more uniform the droplet size and consequently, the more stable. Values between 2 and 5% are good indicators of stability. Higher values may indicate susceptibility to instability phenomena such as flocculation and coalescence.^66,90^ Among the techniques that can be used to verify polydispersity, hydrodynamic diameter and zeta potential, Dynamic Light Scattering (DLS), also known as photon correlation spectroscopy (PCS), is the most used due to the ease of obtaining results compared to other techniques, without the need for prior sample preparation. This technique uses fluctuations in the intensity of scattered light in the sample to determine the diffusion coefficient, relating it to the hydrodynamic radius.^91–96^

### 4.4 Accelerated Stability

Preliminary stability tests should be performed at the beginning of the formulation process of a cosmetic product and serve to accelerate possible instabilities that may occur in the systems or signals that need attention, in addition to contributing information related to product safety.^96^

The centrifuge test is used to verify the occurrence of instability phenomena such as flocculation, coalescence, or creaming. The evaluation considers macroscopic and microscopic aspects of phase separation. For the thermal stress and freeze–thaw cycle tests, the parameters evaluated depend on the characteristics of the product, but in general, parameters such as appearance, color and phase separation characteristics are evaluated. pH and conductivity are used to measure the passage of electric current through the system, and changes can indicate instability. Increases in conductivity may be related to coalescence, and decreases may be related to flocculation.^96^ The normal pH of the skin is slightly acidic, varying between 4.6 and 5.8. Maintaining these values is important for maintaining the integrity of the skin, ensuring bactericidal and fungicidal protection as well as maintaining the activity of enzymes involved in the production of ceramides.^97,98^ Values that are too high can be indicative of bacterial growth or the occurrence of chemical reactions, which can compromise the final product.^96^

### 4.5 Thermogravimetric Analysis (TG) and Differential Scanning Calorimetry (DSC)

It is an analytical technique used to monitor the physical and chemical changes of a sample when exposed to controlled heating. Various factors influence these mass changes (loss or gain), from the evaporation of volatile compounds, moisture loss, gas desorption, water absorption or loss, heterogeneous chemical reaction, or thermal decomposition in an inert atmosphere. The mass variation is monitored and evaluated as a function of temperature or exposure time and presented in a curve.^91^

DSC, a thermoanalytical technique, is used to measure the difference in the amount of heat required to raise the temperature of a sample relative to a reference; therefore, the reference sample must have a well-defined thermal capacity. This approach is useful for identifying phase transitions and analyzing the proportion of solid lipids in the system, as well as monitoring possible crystallization of oils or surfactants, which can influence the stability of nanoemulsions,^99^ as well as providing indirect information about material behavior. The technique is based on the structural changes manifested by the sample under the action of temperature, and the obtained curve allows the determination of enthalpy changes (ΔH) as a function of time or temperature. This indicates that the sample temperature becomes lower in endothermic processes and higher in exothermic processes.^91^

### 4.6 Penetration and Release Tests

The effectiveness of nanoemulsions in cosmetic applications relies heavily on their ability to enhance the transdermal delivery of bioactive compounds. Due to their small droplet size (20–200 nm) and high surface area, nanoemulsions facilitate penetration through multiple skin pathways, overcoming the barrier function of the stratum corneum (SC).^100–103^

Follicular penetration is a primary route for nanoemulsions, particularly in hair-bearing skin areas. The small droplet size allows accumulation in hair follicles, which act as reservoirs for prolonged release of actives such as flavonoids and lipophilic vitamins. Studies using confocal laser scanning microscopy (CLSM) have shown that O/W nanoemulsions loaded with quercetin penetrate up to 300 μm deeper via follicular routes compared to conventional emulsions.^100–103^

Intercellular and transcellular pathways are enhanced by the flexible nature of nanoemulsion droplets, which temporarily disrupt lipid packing in the SC. Surfactants with optimal HLB values (e.g., Tween 80, lecithin) reduce interfacial tension, promoting fluidization of corneocytes and enabling diffusion of lipophilic compounds like beta-carotene and vitamin E.^100–103^

Controlled release kinetics are governed by droplet composition and emulsifier type. Low-energy nanoemulsions (e.g., phase inversion) exhibit zero-order release profiles, ideal for sustained antioxidant delivery in antiaging products. In contrast, high-energy systems (ultrasonication) favor burst release, suitable for immediate skin brightening with vitamin C.^100–103^

Franz diffusion cell studies (Figure 11) using human or porcine skin equivalents remain the gold standard for evaluating permeation. Recent reports indicate that nanoemulsions increase the skin retention of phenolic compounds by 3–6 fold compared to free forms, with minimal systemic absorption. Permeation enhancers (e.g., terpenes, fatty acids) can be coencapsulated to further modulate SC solubility parameters.^100–103^

**Figure 11:**
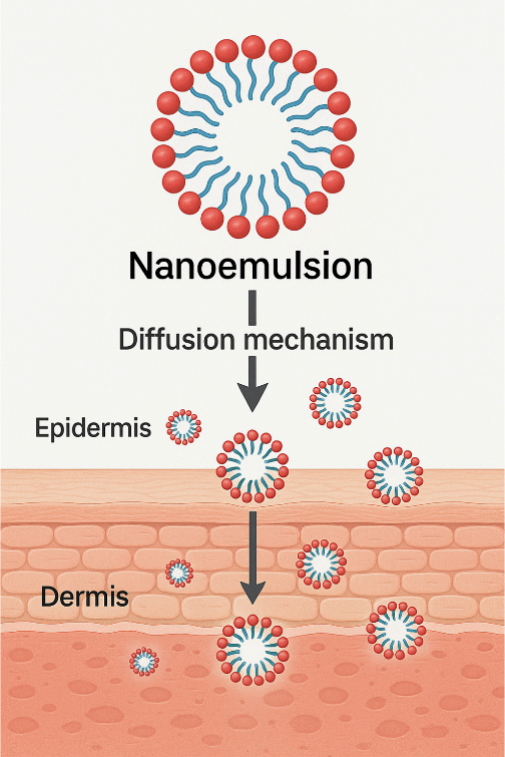
Nanoemulsion skin penetration and controlled release.

In vivo tape stripping and Raman spectroscopy confirm deeper dermal targeting, with nanoemulsions achieving up to 40% higher deposition in the viable epidermis than microemulsions. This is critical for bioactives targeting oxidative stress, such as resveratrol and curcumin.^100–103^

Future prospects include stimuli-responsive nanoemulsions (pH, temperature, or enzyme-triggered) for on-demand release in damaged skin, and hybrid systems combining nanoemulsions with microneedles or iontophoresis for enhanced delivery in scar tissue or photoaged skin.^100–103^

### 4.7 Efficacy Evaluation

Clinical studies and sensory evaluations are essential components for determining the efficacy of cosmetic products containing nanoemulsions. Sensory evaluations, in particular, have proven effective in analyzing consumer perceptions of attributes such as spreadability, oiliness, and skin absorption. One study showed that nanoemulsions containing encapsulated lipoic acid were preferred by consumers due to their lower stickiness and lower olfactory residue compared to the nonencapsulated version.^104^

In addition, nanostructured formulations demonstrated greater penetration of active ingredients into the skin, which was confirmed by ex vivo studies on pig skin and in vitro analyses. These formulations have been analyzed for clinical efficacy in trials that measure penetration and skin retention of cosmetic active ingredients.^105^

## 5 Bioactive Compounds and Their Relevance in Cosmetics

Bioactive compounds, such as antioxidants and anti-inflammatories, play a crucial role in protecting the skin from oxidative damage and combating premature aging (Figure 12). They neutralize free radicals, which are unstable molecules responsible for the degradation of skin cells, which can lead to conditions such as skin cancer and premature aging.^106^ Encapsulating these compounds in nanoemulsions increases their stability and efficacy, ensuring controlled release and protection against degradation by light and oxygen.

**Figure 12:**
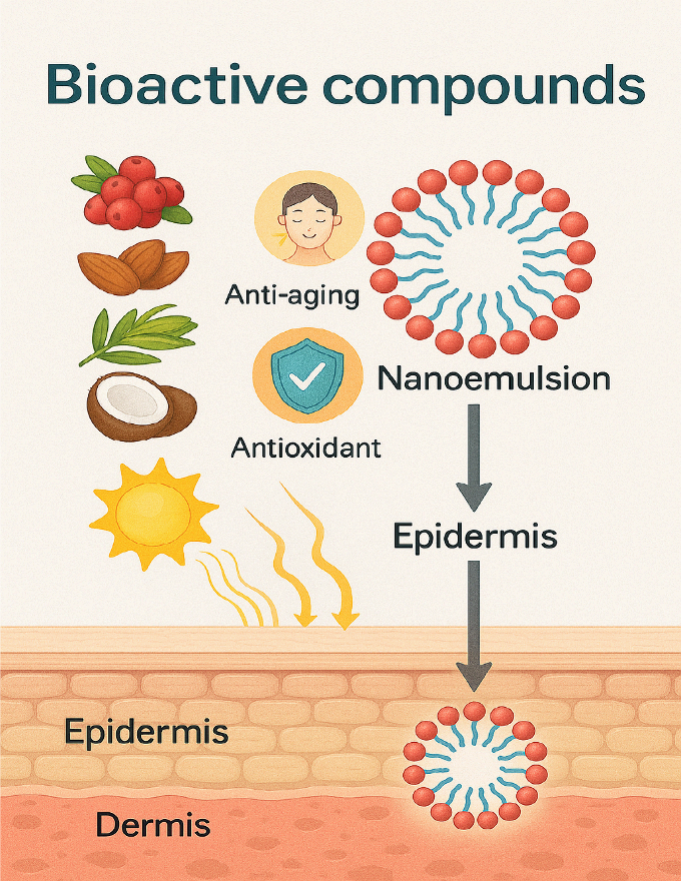
Nanoemulsion encapsulation: protection and controlled release of bioactive compounds.

It is possible to verify that recent studies address the investigation of bioactive compounds from natural sources and their possible applications in cosmetics, such as a study by Rajaei et al.^107^ investigated the biological activities and revealed the presence of bioactive compounds such as flavonoids, polysaccharides and polyphenols in jujube byproducts (Ziziphus jujuba) while Zhu et al.^108^ presented health benefits in the protection of the intestinal barrier from cellular models. The effect of extracts of bioactive compounds from Canthium horridum blume leaves using polyols on the skin was evaluated in the study by Myo and Khat-Udomkiri^109^ where the main antioxidant bioactive compounds included 4-(butoxymethyl) phenol, 3-O-caffeoyl-4-O-methylquinic acid, 3-(2G-glucosylrutinoside) quercetin, 2,4-dihydroxybenzoic acid. The study revealed efficacy in relation to melanin content, which suggests a potential for application in whitening and antiaging products. Andrade et al.^110^ evaluated the effects of microencapsulation of hydroalcoholic extracts of cashew (Anacardium occidentale L.) stalks and pomace, verifying antioxidant activities as well as the maintenance of phenolic content during storage. Halim et al.^111^ evaluated the functional properties of coconut water, showing high antioxidant and antiaging capacities, making it possible to use it in cosmetics.

Bioactive compounds encapsulated in nanoemulsions offer significant benefits to the skin, including improving hydration, preventing damage caused by UV radiation, and promoting cell regeneration. Studies show that nanoemulsions increase the bioavailability of these compounds, facilitating deep penetration into the skin and providing a sustained action over time.^112^ Furthermore, nanoemulsion formulations containing antioxidants such as ascorbic acid have shown significant effects in reducing wrinkles and hyperpigmentation, promoting cell renewal and skin lightening.^113^

### 5.1 Antioxidants

Antioxidants are molecules capable of interacting with free radicals and interfering in the oxidation process, oxidizing in place of other important molecules in the body.^11,114^ These molecules can act by donating electrons to reactive species or as metallic chelators. They are widely used for the conservation and stabilization of foods, beverages, medicines, and beauty products,^114^ for the prevention of lipid rancidity, and as active ingredients in cosmetics as well as in food supplementation.^115–118^

Antioxidants can be subdivided into endogenous (also known as enzymatic and nonenzymatic) and exogenous, which can be further categorized as natural (obtained through the diet) or synthetic.^119^ Figure 13 summarizes antioxidant mechanisms and applications.

**Figure 13:**
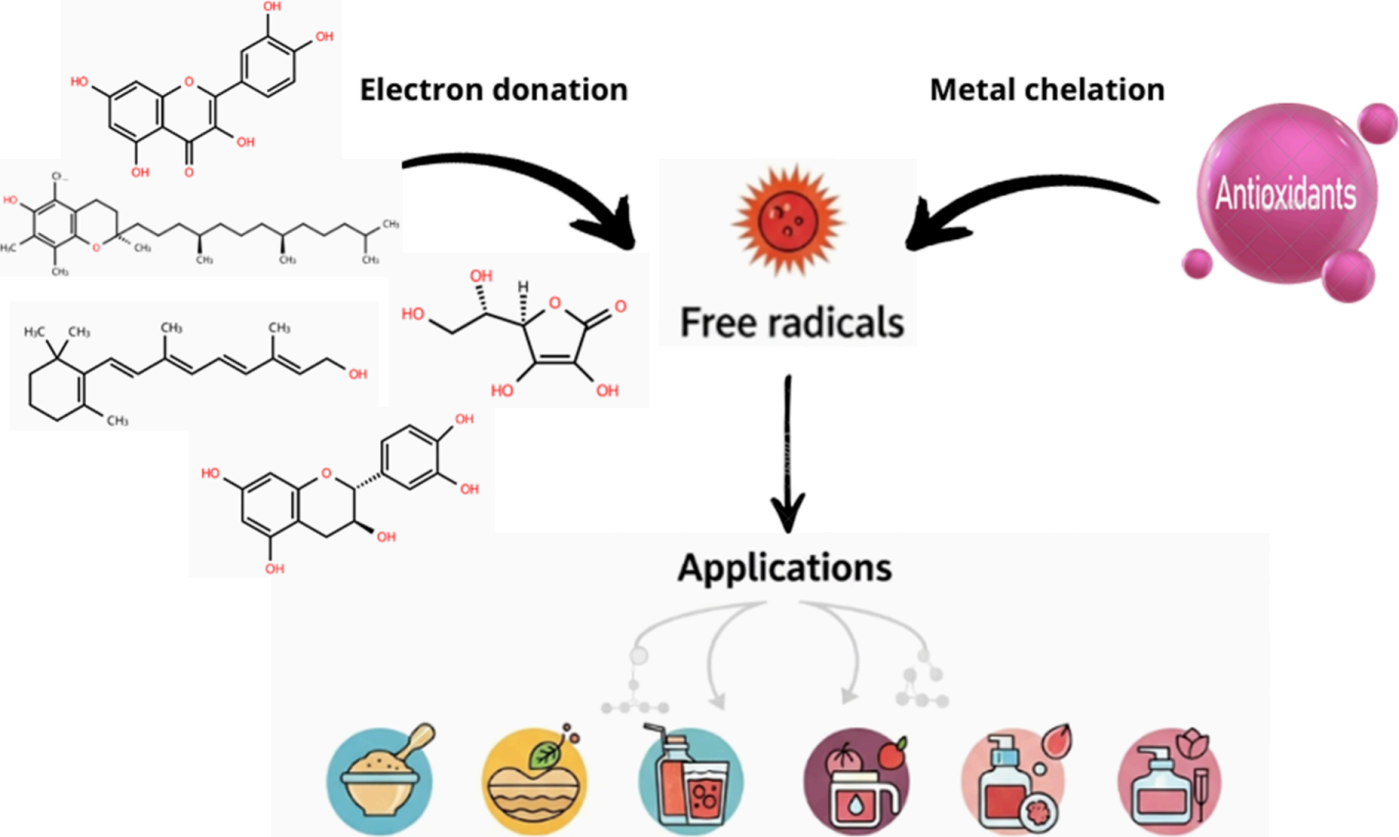
Antioxidants: mechanisms and applications.

#### 5.1.1 Synthetic Antioxidants

Synthetic antioxidants are artificially produced compounds that are crucial in preventing oxidation in various products.^120^

Regarding the function of these antioxidants, their main role is to prevent the oxidation of lipids, proteins, and other oxidation-sensitive components. They act by neutralizing free radicals, highly reactive molecules that can damage cells and tissues, leading to product degradation. Lipid oxidation, for example, results in rancidity, affecting the taste and smell of food and generating harmful compounds.^121,122^

As for industrial applications, in the food industry, synthetic antioxidants such as BHA (butylated hydroxyanisole), BHT (butylated hydroxytoluene), and TBHQ (*tert*-butylhydroquinone) are widely used to preserve processed foods, oils, and fats, preventing them from becoming rancid. They help maintain the nutritional value and safety of food during extended storage.^120^

In the cosmetics industry, synthetic antioxidants are used to stabilize formulations and prevent the oxidation of active ingredients, ensuring the efficacy and safety of beauty products such as creams, lotions, and sunscreens.^11^

In the pharmaceutical industry, synthetic antioxidants are added to medications to protect active ingredients from oxidative degradation, ensuring the potency and stability of products throughout their shelf life.^123^

Furthermore, these compounds are also used in plastics, rubbers, and fuels to prevent degradation by oxidation, prolonging the durability and efficacy of these materials.^124^

#### 5.1.2 Natural Antioxidants

Natural antioxidants are compounds found in foods and plants that protect the body’s cells against damage caused by free radicals and oxidative processes. These compounds are essential for maintaining health and help prevent chronic diseases such as cancer, heart disease, and neurodegenerative disorders.^125,126^

The most common natural antioxidants include vitamin C, found in citrus fruits and bell peppers, vitamin E, present in nuts, seeds, and vegetable oils, and beta-carotene, which is in carrots, pumpkins, and sweet potatoes. Other important antioxidants are polyphenols and flavonoids, which are found in berries, green tea, dark chocolate, and legumes.^127^

These antioxidants neutralize free radicals and unstable molecules that can damage cells and DNA, contributing to premature aging and the development of diseases. Additionally, they help reduce inflammation and improve immune function.^128^

#### 5.1.3 Mineral Antioxidants

Mineral antioxidants are essential inorganic elements that protect cells against oxidative damage caused by free radicals. They play a vital role in maintaining overall health and preventing various chronic diseases.^129,130^

The main mineral antioxidants include selenium, zinc, copper, and manganese. Selenium is part of antioxidant enzymes like glutathione peroxidase, which protects against oxidative stress. Rich sources of selenium include Brazil nuts, seafood, meat, and eggs. Zinc is crucial for the functioning of enzymes like superoxide dismutase (SOD), which neutralizes superoxide free radicals, and is found in red meat, poultry, nuts, and legumes. Copper is also a component of SOD and is obtained from seafood, nuts, seeds, and liver. Manganese, another component of SOD, is found in whole grains, nuts, legumes, and tea.^131–133^

These minerals are indispensable for the body’s antioxidant defense, neutralizing free radicals and protecting against cellular damage and inflammation. Additionally, they aid in DNA repair and regulate the immune system. Adequate intake of these materials is essential to prevent diseases related to oxidative stress, such as heat disease, cancer, and neurodegenerative diseases.^134,135^

### 5.2 Phenolic Compounds

Phenolic compounds are a diverse class of chemical compounds found abundantly in plants, known for their antioxidant properties and a wide range of potential health benefits for humans. They are characterized by the presence of one or more aromatic rings with one or more hydroxyl groups attached, giving them the ability to neutralize free radicals and other oxidizing agents.^136–138^

These compounds are classified into several subclasses, including flavonoids, tannins, phenolic acids, and lignans, among others. Each class has distinct chemical structures that determine their specific biological properties. For example, flavonoids are extensively studied for their antioxidant, anti-inflammatory, and potential protective effects against chronic diseases such as cancer and heat disease. They are found in fruits, vegetables, tea, and wine, significantly contributing to the health benefits associated with consuming these foods.^139–141^

Tannins, on the other hand, are known for their astringent properties and are found in fruits like grapes and apples, as well as in teas and red wines. They possess antimicrobial activity and can aid in digestive health. Phenolic acids, such as gallic acid and caffeic acid (Figure 14), are found in foods like coffee, fruits, and whole grains, demonstrating antioxidant and anti-inflammatory activities that can be beneficial for cardiovascular and metabolic health.^3,142,143^

**Figure 14:**
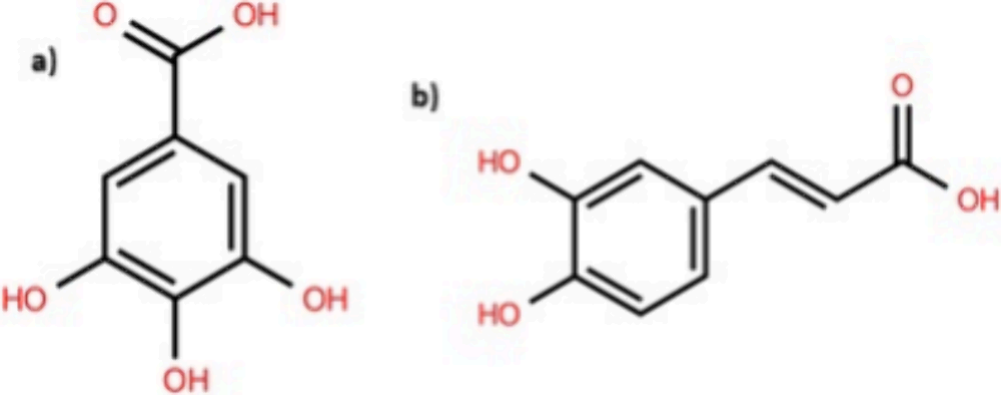
Molecular structures. (a) Gallic acid; (b) caffeic acid.

Phenolic compounds are often produced in response to environmental stressors. Responsible for sensory characteristics such as color, bitterness, astringency, and flavor in food, these compounds play a crucial role in protecting against lipid oxidation. Their chemical structure, comprising an aromatic ring with hydroxyl groups, allows for a variable composition that enables a wide range of biological activities. Notably, they act as potent antioxidants by scavenging free radicals in the body, making them important in promoting health and preventing diseases related to oxidative stress.^144^

In addition to their antioxidant properties, phenolic compounds have been associated with benefits such as improved immune function, reduced risk of neurodegenerative diseases, and modulation of the intestinal microbiota. Studies continue to explore their potential effects in the prevention and treatment of a variety of health conditions.^145,146^

#### 5.2.1 Flavonoid Compounds

Flavonoids are a widely researched group of natural polyphenolic compounds found in various plants and recognized for their antioxidative, anti-inflammatory, and photoprotective properties, which make them valuable in cosmetic formulations. However, challenges such as low bioavailability and rapid degradation limit their effectiveness when applied topically or ingested. To address these issues, nanoemulsions have emerged as an effective delivery system, enhancing the stability and bioefficacy of flavonoids in skincare applications. For example, flavonoid nanoemulsions have shown increased skin penetration and sustained release, which contribute to improved antioxidant activity and prolonged skin benefits.^147^ Incorporating flavonoids into nanoemulsions thus enables a reduction in oxidative damage and supports skin health in cosmetic applications.^148^

Building upon the increased bioavailability and skin stability of flavonoid nanoemulsions, further advancements highlight the use of natural emulsifiers and innovative encapsulation strategies to maximize therapeutic effects and reduce skin sensitivity. By utilizing proteins and lipids in the nanoemulsion formulation, studies have demonstrated that flavonoid compounds can achieve sustained release and deeper skin penetration, enhancing their anti-inflammatory and photoprotective benefits for sensitive skin types. This approach not only improves flavonoid stability but also aligns with consumer preferences for natural and biodegradable ingredients.^149^

#### 5.2.2 Anthocyanins

Anthocyanins, a type of flavonoid responsible for the vibrant colors in many fruits and flowers, are increasingly used in cosmetics due to their potent antioxidant and anti-inflammatory properties. These compounds can help combat photoaging and improve skin elasticity. However, anthocyanins face stability issues due to their sensitivity to pH, light, and temperature, which can be mitigated by nanoemulsification. Recent studies show that nanoemulsions can protect anthocyanins from degradation and improve their bioactivity when applied to the skin.^150^ This encapsulation not only enhances stability but also facilitates better penetration into the skin layers, thereby maximizing anthocyanin’s beneficial effects on skin tone and inflammation reduction.^150^

Expanding on the stability provided by nanoencapsulation, recent research has refined the use of anthocyanins in skincare through innovative delivery systems that boost penetration while preserving antioxidant properties. For instance, anthocyanin nanoemulsions prepared from blueberry extracts have shown enhanced bioavailability, reduced irritation, and significant antioxidant stability in skin care applications, underscoring their effectiveness in antiaging formulations.^151^ Additionally, complex nanocarriers such as protein-based systems further extend anthocyanin bioactivity by enhancing cellular uptake and resilience under environmental stresses, solidifying their value in cosmetics.

#### 5.2.3 Carotenoids

Carotenoids are lipid-soluble antioxidants that are effective in protecting skin cells from oxidative stress and UV-induced damage, making them highly suitable for antiaging cosmetic products. Despite their benefits, carotenoids suffer from poor solubility and instability, which limits their use in cosmetics. Nanoemulsion technology addresses these limitations by enhancing the solubility and stability of carotenoids, thus improving their availability and effectiveness upon topical application. Studies have shown that carotenoid-loaded nanoemulsions retain high antioxidant activity and offer superior UV protection when compared to conventional formulations.^152^ This encapsulation not only improves the functional properties of carotenoids but also makes them a viable option for skincare products that target aging and skin health.^153^

In addition to their potent antioxidant properties, nanoemulsified carotenoids have shown promise in protecting skin from UV radiation and oxidative stress. By encapsulating carotenoids in nanoscale carriers, researchers have significantly increased their stability and bioavailability, allowing these compounds to provide prolonged protective effects on the skin. This encapsulation technique, which mitigates carotenoids’ sensitivity to light and temperature, ensures their efficacy in antiaging and sun-protective formulations, presenting a viable alternative to synthetic UV filters.^153^

#### 5.2.4 Non-flavonoid Compounds

Beyond flavonoids, nonflavonoid polyphenols such as phenolic acids and tannins also exhibit significant antioxidative and antimicrobial effects, which are advantageous for cosmetic applications. Their incorporation into nanoemulsions can enhance these bioactive compounds’ stability and skin penetration. Phenolic compounds like caffeic acid, when encapsulated in nanoemulsions, have demonstrated improved efficacy in reducing inflammation and oxidative damage in skin cells. Additionally, nanoemulsions facilitate the slow release of these nonflavonoid compounds, thus extending their beneficial effects over time.^99^ By leveraging nanoemulsion technology, nonflavonoid compounds can be effectively incorporated into skincare products to offer enhanced antioxidant protection and support overall skin health.^154^

Following the benefits observed in flavonoid nanoemulsions, nonflavonoid compounds such as phenolic acids have also shown enhanced effects when incorporated into nanoemulsions, particularly for anti-inflammatory and antimicrobial uses. By enabling a controlled release mechanism, nanoemulsions of these compounds provide sustained activity and increased bioavailability, addressing limitations associated with traditional formulations. These advancements improve the compatibility of phenolic compounds with cosmetic applications, offering a prolonged, gentle antioxidant effect that suits sensitive and damaged skin.^155^

### 5.3 Vitamins

Vitamins play a fundamental role in the development of living beings. Among the vitamins obtained from plants, they can be further subdivided into vitamin A (retinol) and vitamin E (tocopherols and tocotrienols) as fat-soluble, and vitamin C (ascorbic acid) as water-soluble.^156^

Vitamin A, in addition to participating in cell growth and differentiation, is essential in maintaining the integrity of the cells that make up the skin,^156^ and contributes to the production of type I collagen, elastin, and fibronectin. In the study by Sadik et al.,^157^ improvement was observed in the pigmentation of spots, wrinkles, fine lines, shine, and pore size when 3% of the active ingredient was applied for 6 weeks.

Vitamin A, an essential fat-soluble micronutrient, supports various biological functions, including vision, immune defense, and cellular growth and differentiation. It exists primarily in two forms in the diet: preformed vitamin A (retinol and retinyl esters) from animal products, and provitamin A carotenoids from plant sources like beta-carotene. Preformed vitamin A is absorbed and utilized more efficiently than carotenoids, which must be converted in the body to active forms. These active forms, such as retinoic acid, regulate gene expression through interactions with nuclear receptors, impacting processes like cellular differentiation and immune responses.^158^ Due to its bioavailability challenges and sensitivity to oxidation, vitamin A is prone to degradation from exposure to light, heat, and oxygen, posing formulation challenges for its incorporation into cosmetics.^159^

In cosmetics, vitamin A derivatives like retinyl palmitate are commonly used for their antiaging effects, attributed to retinoic acid’s role in enhancing cell turnover and collagen synthesis. However, maintaining vitamin A stability in formulations is complex, and nanoencapsulation has emerged as a promising solution. Nanoemulsions protect vitamin A from oxidative degradation, extending its efficacy in skin products. Furthermore, encapsulation methods involving amino acids or starch derivatives can enhance stability and bioavailability, ensuring the vitamin’s therapeutic effectiveness upon topical application. Nanoemulsion systems, therefore, present an advanced, stable vehicle for vitamin A in cosmetics, maximizing skin absorption while minimizing degradation.^158,159^

In the study of Yang et al.,^160^ the performance of a nanoemulsion developed on the basis of hydrophobically modified inulin for greater stability and transdermal delivery of retinyl propionate was investigated, observing a small droplet size (<100 nm) and high physical stability of the nanoemulsions, in addition to a high retention rate (greater than 80%), and high transdermal delivery, in the epidermis and dermis, from in vitro tests, by Franz diffusion cell, compared to conventional emulsions. Yousefi et al.^161^ optimized the conditions for developing multiple W/O/W nanoemulsions containing retinol and sesamol using different concentration of tween 80, and span 80, obtaining of 92.93% encapsulation efficiency and particle size of 381.94 nm.

Vitamin C prevents lipid peroxidation and scavenges ROS.^138^ It is associated with cosmetics as an agent related to skin brightening, antioxidant activity, antiaging, and anti-inflammatory action.^160^ When a cosmetic containing 20% vitamin C was used by women every day in a study, increased elasticity, shine, smoothness of wrinkles, and color improvement were observed.^162^ Vitamin C, also known as ascorbic acid, is a weak organic acid with water-soluble antioxidant with a vital role in the body. Many plants and animals can synthesize this vitamin from glucose. Still, it cannot be synthesized naturally by humans and some vertebrates because they do not have the L-gulono-1,4-lactone oxidase gene, which encodes one of the enzymes responsible for the ascorbic acid biosynthesis; it is necessary to obtain it through food, from vegetables and fruit, which are the primary sources of this vitamin.^163,164^

The best-known sources of vitamin C are fruits such as Kakadu plums, Australian plums, guavas, gooseberries, kiwi fruit, strawberries, green leafy vegetables such as kale, spinach, peppers, tomatoes, asparagus, Brussels sprouts, acerola and rose hips. Grains, roots, and tubers have deficient concentrations of this vitamin.^165^

The chemically active and commonly used form is l-ascorbic acid. This structure determines its physical and chemical properties like high solubility and easy degradation under exposure to light, oxygen, and temperature changes.^166^ There is great interest in synthesizing active and chemically stable molecules due to the instability of ascorbic acid in nature. Another critical aspect of its structure is its action in the aqueous compartments of cells due to its water-solubility.^167^

Among the applications of vitamin C, it is possible to highlight its participation in enzymatic reactions, maintenance of the skin and blood vessels, regulation of the immune response, and aid in the absorption of iron, in addition to the well-known prevention of scurvy.^168^ Thanks to its antioxidant properties, vitamin C also helps prevent and treat chronic diseases such as diabetes, macular degeneration, glaucoma, cataracts, heart disease, atherosclerosis, stroke, and cancer, as well as being used in the chemical, food, and cosmetic industry.^165^

Vitamin E is also known for its antioxidant activity, preventing lipid peroxidation and participating in collagen synthesis.^6^ In addition to its use in cosmetic products to maintain the skin’s natural skin barrier, reports show its use in sun protection formulations.^31,67,169^ Vitamin E is exclusively obtained from plants, therefore, all means of supplementation for the body come from food. It can be obtained from various oils (wheat germ, sunflower, safflower, soybean, corn, cottonseed, palm), nuts, and cereal products.^6^ The antioxidant mechanism of vitamin E primarily occurs through its ability to donate phenolic hydrogens (H+) to free radicals.^170^

Vitamin E refers to a group of lipophilic compounds formed by an aromatic ring to a side chain.^171^ When the chain is in a saturated form, the tocopherol isomers (alpha, beta, gamma, and delta-tocopherol) are obtained. Three of the carbons in the chain are asymmetric, allowing for the existence of eight stereoisomers.^172,173^ When the chain is in an unsaturated form, with three conjugated double bonds forming an isoprenoid chain,^15^ the tocotrienol isomers (alpha, beta, gamma, and delta-tocotrienol) are obtained. The isomers are differentiated by the position of the methyl and phenol groups on the aromatic ring.^171^

The most common and biologically active form of vitamin E in nature is RRR-α-tocopherol. This bioavailable form tends to accumulate in environments with higher free radical production, such as in the endoplasmic reticulum of the lung, heart, and mitochondrial cells.^171,173^

Although the isomers exhibit antioxidant activity, this capacity varies among each isomer and decreases in the following order: β and γ-tocopherol, with reduced activity of 15 to 30%, and δ-tocopherol, which is practically inactive.^15,173^ Additionally, tocotrienols are suggested to be potential neuroprotective agents following ischemic stroke.^174^

Studies also highlight the role of vitamin E in degenerative diseases such as Alzheimer’s,^175^ in preserving cardiac function during ischemic and reperfusion injuries,^176^ in cardioprotective efficacy when administered with apelin,^177^ and in the treatment of certain types of cancer.^178^

### 5.4 Challenges in the Incorporation and Stabilization of Bioactive Compounds in Cosmetic Products

Incorporating bioactive compounds into cosmetic products presents several challenges related to their stability and efficacy. Many bioactive compounds, such as polyphenols, vitamins, and peptides, are sensitive to environmental factors like heat, light, and oxygen, which can lead to degradation and reduced effectiveness. Stabilization methods, such as encapsulation, are essential to protect these compounds, but ensuring that the bioactive molecules remain active and bioavailable within the cosmetic formulation is difficult. For instance, conventional emulsification techniques often struggle with maintaining consistent droplet size and stability in the face of oxidation or other degradative processes.^179^ Additionally, some preservatives and surfactants commonly used in cosmetics can have toxic or sensitizing effects, making it necessary to balance safety with the need for effective stabilization.^179^

#### 5.4.1 Thermodynamic Stability

One of the key challenges in incorporating bioactive compounds into cosmetics is ensuring their thermodynamic stability. Many bioactive molecules, such as phenolics, vitamins, and peptides, tend to be thermodynamically unstable when exposed to factors such as heat, UV light, and oxidation. This instability can lead to a loss of bioactivity, reducing the overall efficacy of the cosmetic product. For example, studies show that thermal degradation of bioactive compounds can occur at elevated temperatures, such as during the production process, leading to the breakdown of critical ingredients like antioxidants and polyphenols.^179^

The process of stability refers to a system’s ability to resist chemical or physical changes in the face of changing conditions, and can be differentiated into kinetic and thermodynamic stability. Because they are made up of small droplets, nanoemulsions are not affected by gravity, but they are influenced by Brownian movement (particle movement), which is responsible for advancing or delaying instability processes in nanoemulsions. In this sense, kinetic stability is related to the movement of the particle itself, where a stable system shows resistance to undergoing reactions or transformations over time, while thermodynamic stability is related to the chemical balance of the system’s constituents, which can lead to a reduced state of energy.^180,181^ Therefore, a system can have only kinetic and thermodynamic stability, or combinations of stability, so it is necessary to understand these parameters in order to develop systems that are stable in one or both cases.

Thermodynamic stability is directly related to the tendency of a system to maintain the lowest energy state at equilibrium. Reactions that produce thermodynamically stable products reduce the system’s free energy, allowing it to maintain this state. According to the Second Law of Thermodynamics, in irreversible processes, entropy always increases, indicating that a stable system reaches maximum entropy under certain conditions. Thus, to be thermodynamically stable, a system must be in a favorable state in terms of free energy and entropy, minimizing its potential for spontaneous changes. In the case of nanoemulsions, the phases are made up of two immiscible substances, each with different thermodynamic properties. When these phases come together, a process of repulsion occurs, generating a tension between them that makes mixing difficult. To preserve thermodynamic stability, the phases tend to maintain a small area of contact called the interface, which Gibbs compared to a dividing line.^182^

Starting from the Gibbs free energy, the parameters that are directly related to the thermodynamic stability of nanoemulsions during the formation process can be calculated. The equations that describe these behaviors are shown in Table 3.^182^

**Table 3:** Parameters Directly Related to the Thermodynamic Stability of Nanoemulsions during the Formation Process

Equation	Description	Application	Mean of terms
G = U+pV–TS	Gibbs energy free	Used to understand the relationship between internal energy and entropy during emulsification, essential for assessing the stability of the system.	G: free energy;
			U: internal energy;
			p: pressure;
			V: volume;
			T: absolute temperature;
			S: entropy.
ΔG = ΔU+pΔV+VΔp–TΔS	Change in Free Energy: Relates the changes in internal energy, volume, pressure and entropy during emulsification.	Describes how the change in free energy relates to the process of transformation of two separate phases in an emulsion, which is fundamental for assessing the spontaneity of the process.	ΔG: change in free energy;
			ΔU: change in internal energy;
			pΔV: work of expansion;
			VΔp: work of pressure;
			TΔS: entropy variation.
ΔG = (ΔU+pΔV)–TΔ*S*= ΔH–TΔS	Free Energy under Constant Conditions: Relates enthalpy (ΔH) to the change in free energy.	It is important to understand how the internal energy of the system changes during the formation of the emulsion, considering that the pressure and composition are constant during the formation, the enthalpy is equal to the change in internal energy, which is equal to the work.	ΔH: change in enthalpy; the other terms remain the same.
ΔGform = ΔW–TΔS	Free Energy of Formation: Considers the work done to form the emulsion and the entropy of the system	calculate the free energy involved in the formation of the emulsion, taking into account the energy needed to reduce the size of the droplets.	ΔW: work done; the other terms remain the same.
ΔW = γΔA	Work as a Function of Interfacial Tension: The work is expressed by the interfacial tension (γ) and the change in interface area (ΔA).	Work can be written as a function of interfacial tension and surface area change	ΔW: work done;
			γ: interfacial tension;
			ΔA: change in interface area.
ΔGform = γΔA–TΔS	Total Change in Free Energy of Formation: The total surface area increases as the droplet size decreases. The dispersion of the oil droplets increases the disorder in the system.As entropy increases, the ΔG term becomes negative	It evaluates the total change in the free energy of emulsion formation, highlighting how the reduction in droplet size and the increase in disorder contribute to the thermodynamic favorability of nanoemulsion formation.	ΔGform: change in the free energy of formation; the other terms remain the same.

Knowing that one of the keys to the development of emulsions is achieved by reducing interfacial tension, the use and proper choice of surfactants contributes directly to the success of the formulation, avoiding instability phenomena, in addition to reducing tension, surfactants provide kinetic stabilization and prevents particle aggregation through protective coatings around the droplets and steric/electrostatic repulsion. This combination results in highly stable nanoemulsions that are resistant to destabilizations such as coalescence and flocculation, although they can be affected by Ostwald ripening over time. By choosing the right type of oil and emulsifier, kinetic stability can be prolonged for months.^58,182^

#### 5.4.2 Enhancing Stability through Encapsulation

To mitigate these stability issues, encapsulation technologies are increasingly being used. These technologies can protect bioactives from harsh conditions like high temperatures or pH changes, thereby improving their thermodynamic stability. For instance, encapsulating bioactive compounds in emulsions or nanoemulsions can significantly enhance their resistance to degradation during storage and use. The choice of emulsifiers and wall materials plays a critical role in this process. Research has shown that using suitable encapsulation techniques, such as ionic gelation or microencapsulation, helps improve thermal stability by isolating the bioactive compounds from the external environment, leading to improved longevity of the products.^183^

#### 5.4.3 Impact of Temperature and Rheological Properties

Temperature fluctuations can impact the thermodynamic stability and overall functionality of bioactive ingredients. Rheological analysis of cosmetic formulations highlights how emulsifiers and stabilizers help maintain consistent viscosity and product texture even under varying thermal conditions. This ensures the kinetic stability of emulsions, which is essential for their longevity and efficacy in cosmetic products.^184^ High temperatures typically accelerate the degradation of bioactive compounds; however, formulations designed with appropriate emulsifiers and stabilizers can maintain product integrity over time.

## 6 Applications in Cosmeceuticals

Nanoemulsions offer significant benefits for the delivery of bioactive compounds in cosmetic applications. Their submicron droplet size enhances the solubility of hydrophobic (water-insoluble) compounds, making them more accessible for absorption and increasing bioavailability. The kinetic stability of nanoemulsions prevents issues such as coalescence, flocculation, and sedimentation, which are common in conventional emulsions, thus prolonging the shelf life and efficacy of the products.^185^ Additionally, nanoemulsions enable controlled release of encapsulated bioactives, enhancing their stability and protecting them from environmental factors, which is particularly valuable in the formulation of cosmetics targeting sustained effects.^185^

Building on the advantages of nanoemulsions in increasing bioavailability and solubility, their high surface area-to-volume ratio also supports better interaction with the skin, which is essential in cosmetic applications where consistent efficacy over time is desired. Nanoemulsions offer a more even distribution of active ingredients across the skin’s surface, enabling enhanced dermal absorption and improved control over the release rate of bioactives. This controlled release minimizes potential skin irritation by delivering ingredients gradually, which is particularly valuable for sensitive skin formulations. Such qualities make nanoemulsions an ideal solution for incorporating active compounds in antiaging and moisturizing products, where sustained effects and stability are crucial.^185^

Cosmetic formulations increasingly incorporate nanoemulsions due to their enhanced skin penetration capabilities, especially for active ingredients such as antioxidants and UV filters. Studies show that nanoemulsions improve the permeation of these activities, effectively reaching deeper layers of the skin compared to conventional emulsions. This deeper penetration can enhance the efficacy of antiaging, moisturizing, and sun-protection agents in skin-care products. For example, oil-in-water nanoemulsions improve the permeability of nonpolar actives, demonstrating increased absorption and skin retention, leading to better hydration and photoprotection outcomes.^186^

Expanding on nanoemulsions’ impact in cosmetics, their application is now common in products targeting specific skin benefits, such as antiaging, sun protection, and hydration. Recent studies have shown that nanoemulsion-based formulations can significantly enhance the stability and effectiveness of active ingredients like retinoids and vitamins, which are prone to degradation in traditional emulsions. Products with nanoemulsions have demonstrated prolonged bioactivity, meaning users experience longer-lasting effects after application. Additionally, the smaller droplet size of nanoemulsions creates a lightweight, nongreasy feel that is well-suited to consumer preferences, enhancing the appeal and effectiveness of cosmetic products.^186^

### 6.1 Effectiveness in the Delivery of Compounds

There is strong evidence supporting the effectiveness of nanoemulsions in delivering active ingredients. Their small droplet size enhances penetration through the skin barrier, enabling a sustained release of active agents and improving overall efficacy in skincare applications. For instance, CoQ10-loaded nanoemulsions were found to maintain stability and active concentration during storage, offering high retention and controlled release, which is critical for long-lasting effects in antiaging products.^187^ Additionally, formulations using plant-derived antioxidants in nanoemulsions showed enhanced stability and bioactivity, confirming their potential for improved dermal delivery in cosmetic products.^188^

Complementing the advantages of targeted delivery, nanoemulsions have shown efficacy in retaining and releasing active compounds over extended periods, a feature critical for cosmetic applications that rely on sustained ingredient efficacy. Studies reveal that bioactives encapsulated within nanoemulsions retain their effectiveness despite exposure to environmental factors like UV light and oxygen, which often degrade active compounds in standard formulations. This stability underlines nanoemulsions’ suitability for products that require long-term potency, such as sunscreens and antioxidants. Furthermore, their ability to penetrate skin layers supports the deep delivery of active ingredients, optimizing their therapeutic effects and making nanoemulsions a preferred vehicle in modern cosmetic formulations.^187^

## 7 Challenges and Limitations

Emulsions and nanoemulsions are thermodynamically unstable systems, and over time, they tend to separate under external influence or disturbance.^189^ The main physical instability mechanisms are illustrated in Figure 15. Thus, it is known that the correct choice and application of surfactant concentration can directly influence the stability of the system. When nanoemulsions are not properly stabilized, the following physical instability phenomena can be observed:

- **Creaming or sedimentation:** Occurs due to the action of gravity. When the density of the emulsified system is lower than that of the aqueous phase, creaming occurs, causing emulsion during phase separation. When the density is higher, sedimentation occurs, where the emulsified system separates and submerges.^183^
- **Coalescence:** In this process, the interfacial film between the droplets breaks, causing them to merge into larger droplets, leading to phase separation.^183,189^

- **Flocculation:** Known to be a reversible aggregation, it is a process in which the surface layers of the droplets interact, resulting in aggregates.^189^
- **Ostwald ripening:** The main phenomenon affecting nanoemulsions. Characterized by the union of smaller droplets with larger ones, resulting in mass transfer from smaller droplets to larger ones, leading to condensation, increasing particle diameter, and potentially causing phase separation. Rheological properties of the interfacial layers and the presence of surfactants also interfere with the occurrence of this phenomenon.^189^

**Figure 15:**
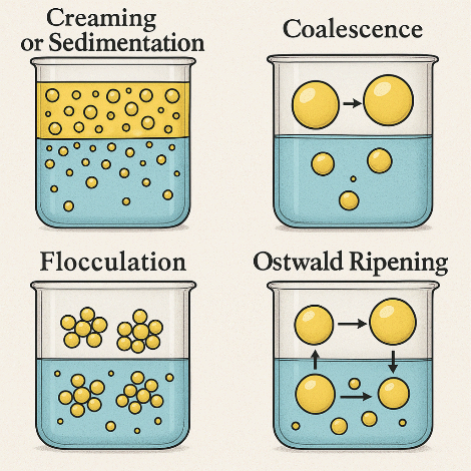
Main physical instability mechanisms in emulsions and nanoemulsions.

It is important to note that the phenomena of flocculation and creaming do not interfere with droplet size distribution, unlike coalescence and Ostwald ripening, which can accelerate the destabilization process of the emulsified system.^189^

### 7.1 Toxicity and Safety for Human Consumption

Nanoemulsions offer a promising vehicle for incorporating bioactive compounds in cosmetics due to their small particle size and increased permeability; however, safety concerns remain a key focus. Studies suggest that nanoemulsions can enable deeper skin penetration, which may elevate exposure to certain active compounds, potentially leading to adverse effects if not properly regulated.^32^ Additionally, bioactive compounds encapsulated in nanoemulsions require toxicity assessments similar to those in traditional cosmetics to avoid risks of bioaccumulation and cytotoxicity.^190^ Despite the limitations of animal testing, advanced in vitro methods can help assess these risks, enhancing the safety profile of nanoemulsion-based cosmetics.^191^

Building on the existing safety assessment, recent studies emphasize the need for advanced testing methods to better understand the systemic impact of nanoemulsions when applied topically or ingested inadvertently. Toxicity profiling through metabolomics and in vitro skin models, such as the SkinEthicRHE model, has shown that while nanoemulsions can enhance the delivery and stability of bioactive compounds, their bioaccumulation potential remains a concern.^191^ The European Union, for example, enforces stringent regulations that restrict certain nanomaterials in cosmetics unless they pass rigorous safety testing. Consequently, as nanoemulsion technologies evolve, adopting standardized toxicity evaluation protocols remains crucial to mitigate risks for long-term human use.^192^

### 7.2 Production Cost on a Large Scale

The production of nanoemulsions on a commercial scale poses notable challenges, primarily due to the requirements for high-quality emulsifiers and energy-intensive processes like high-pressure homogenization and ultrasonication. For instance, replacing synthetic emulsifiers with natural options is a growing trend to align with consumer preferences, but it can increase production costs significantly.^193^ Additionally, achieving the desired nanodroplet size and stability requires sophisticated equipment, which may not be readily accessible to smaller manufacturers. These challenges necessitate innovations to reduce costs while maintaining nanoemulsion stability and effectiveness, making large-scale production a crucial area for ongoing research and development.^194^

Beyond formulation, scaling up nanoemulsion production requires not only equipment but also a careful balance of cost-effective, yet stable, emulsifiers. Studies highlight that while natural emulsifiers provide consumer-friendly alternatives, they can increase costs and may not achieve the same stability as synthetic alternatives under industrial conditions.^193^ Additionally, innovative systems such as microfluidization and high-pressure homogenization are essential for maintaining product quality during scaling, but their high energy demands add to production expenses.^195^ This suggests a need for sustainable advancements in production methodologies to reduce costs while upholding efficacy and consistency.

### 7.3 Regulation and Consumer Acceptance

The regulatory landscape for nanoemulsions in cosmetics is complex, with strict guidelines in regions like the EU, where the safety of nanomaterials is scrutinized intensively before they reach the market. While nanoemulsions allow for innovative product formulations, inconsistencies in global regulations challenge manufacturers, who must meet diverse standards depending on their market. The FDA has recommended rigorous safety testing, although universal definitions for nanomaterials are still lacking.^192^ From a consumer standpoint, acceptance is influenced by a growing awareness of nanotechnology’s benefits and concerns, with many valuing natural ingredients and proven safety above novel formulations. Transparency and evidence-based marketing are thus essential for gaining consumer trust and ensuring widespread adoption of nanoemulsion-based products.^196^

Following the regulatory complexities, consumer acceptance remains a pivotal aspect. As awareness of nanotechnology in cosmetics grows, consumers are increasingly concerned about both the safety and environmental impact of nanoemulsions. Research indicates that transparency in product composition and efficacy, paired with adherence to strict regulatory standards, plays a significant role in fostering consumer trust.^196^ Furthermore, as consumer demand leans toward sustainability, regulations are expanding to include eco-friendly and biodegradable nanoemulsion components, underscoring a holistic approach to product development that appeals to environmentally conscious buyers.^194^

### 7.4 Critical Evaluation of Unresolved Challenges in Nanoemulsions for Cosmetics

While nanoemulsions have demonstrated significant promise in enhancing the incorporation and delivery of bioactive compounds in cosmetics, as evidenced by studies on improved skin penetration^100–103^ and stability of antioxidants like flavonoids and carotenoids,^147–153^ several unresolved challenges persist that limit their widespread adoption. A critical examination of the literature reveals gaps in long-term stability, safety profiles, and scalability, which warrant further investigation.

Regarding stability, many studies highlight kinetic stability advantages due to small droplet sizes (20–200 nm),^53–55,86^ yet real-world instability phenomena such as Ostwald ripening, coalescence, and phase separation remain prevalent during extended storage or under environmental stresses (e.g., temperature fluctuations or pH changes).^89,182–184^ For instance, high-energy preparation methods like ultrasonication^72–74^ often yield stable systems in lab settings, but low-energy approaches (e.g., phase inversion)^73,76–78^ show inconsistent polydispersity and zeta potential in scaled formulations.^66,88,90^ Critically, while natural emulsifiers (e.g., lecithins or proteins)^58–63^ are promoted for biocompatibility,^193^ they frequently underperform compared to synthetic counterparts in preventing aggregation in acidic or high-temperature cosmetic bases.^65,68–70^ Unresolved issues include the lack of standardized protocols for predicting long-term stability across diverse bioactive loads, as current characterization methods like DLS and TG/DSC^91–96^ provide snapshots but fail to model dynamic in-use conditions. Future research should prioritize hybrid emulsifier systems or stimuli-responsive designs to mitigate these limitations.^100–103^

Safety concerns represent another critical gap, particularly with nanomaterials’ potential for dermal absorption and systemic effects.^192,196^ Although nanoemulsions enhance bioavailability,^112,147–149^ studies on cytotoxicity and ecotoxicity are limited and often contradictory.^190,191^ For example, while some reports indicate low irritation for flavonoid-loaded nanoemulsions,^148,149^ others note risks of oxidative stress or allergic responses from surfactants like Tweens.^70,193^ Regulatory frameworks in Europe and North America emphasize nanomaterial labeling and risk assessments,^194,196^ but the literature lacks comprehensive in vivo human trials beyond pilot studies.^104,105,157,162,170^ Notably, the encapsulation of lipophilic actives (e.g., vitamins A, C, E)^156,159–161,159–161,163–178^ reduces degradation but may inadvertently increase follicular penetration, raising questions about long-term genotoxicity or endocrine disruption.^35–38,112^ A more rigorous critique reveals that while green extraction and natural sources are advocated,^11,67,193^ the absence of standardized toxicological models (e.g., integrating metabolomics^191^) hinders reliable safety predictions. Addressing this requires multidisciplinary approaches, including advanced in vitro skin models and longitudinal epidemiological data.

Finally, large-scale production remains a bottleneck, with lab-scale successes (e.g., microfluidics or spontaneous emulsification^72–74,197^ ) not translating efficiently to industrial levels due to high energy costs, equipment scalability, and batch-to-batch variability.^185–189,198^ Critical analysis shows that while food and pharmaceutical applications have advanced,^53,54,198,199^ cosmetic-specific challenges like maintaining sensory attributes (e.g., translucency and rheology)^55–57,105^ are underexplored in production contexts. Economic viability is further compromised by the reliance on expensive natural emulsifiers,^58–63,193^ and environmental impacts from waste in high-energy processes^22–24^ are rarely quantified. Unresolved issues include optimizing continuous-flow systems for cost-effectiveness and sustainability, as suggested by recent reviews.^185,187–189^ Overall, these challenges underscore the need for collaborative efforts between academia and industry to develop predictive modeling tools and eco-friendly protocols, potentially integrating AI-driven optimization for formulation design.

By addressing these gaps, nanoemulsions could evolve from promising lab innovations to robust, market-ready cosmetic solutions, but only if future studies shift from descriptive reporting to hypothesis-driven, comparative evaluations.

## 8 Future Perspectives

The evolution of nanoemulsion-based cosmeceuticals relies on sustainable innovation, predictive technologies, and scalable solutions to overcome current challenges in stability, safety, and large-scale production. Green and biodegradable surfactants, such as rhamnolipids, sophorolipids, and plant-derived saponins, are gaining prominence due to their high emulsifying efficiency, low ecotoxicity, and excellent skin compatibility compared to synthetic alternatives.^58,193,200,201,202^ These biosurfactants support clean beauty trends and reduce environmental impact, with practical applications already demonstrated in Table 2 using moringa oil and bakuchiol in D-phase emulsification systems.^83–85^ Sustainable sourcing of bioactive compounds further strengthens this approach by leveraging agro-industrial byproducts—such as jujube peels, cashew apple pomace, and coconut water—through green extraction techniques including ultrasound-assisted and polyol-based methods.^107–111,165^ This upcycling strategy not only minimizes waste and ecological footprint but also ensures high-purity antioxidants aligned with circular economy principles and ethical supply chains.

Computational modeling is revolutionizing nanoemulsion design by shifting from trial-and-error to data-driven prediction. Molecular dynamics and finite element simulations already forecast droplet coalescence, zeta potential evolution, and transdermal permeation under varying pH, temperature, and shear conditions.^73,89,189,198^ Within this framework, artificial neural networks (ANNs) have emerged as a powerful tool for multivariate optimization and long-term stability forecasting. Recent studies show ANNs accurately predict creaming index and phase separation in virgin coconut oil nanoemulsions using inputs like surfactant concentration, oil viscosity, and homogenization energy.^203^ Similarly, ANN-coupled design of experiments achieved R^2^ > 0.95 in predicting droplet size and rheological behavior of O/W cosmetic nanoemulsions.^204^ Other applications include modeling interfacial tension and phase inversion kinetics for rapid emulsifier screening,^205^ optimizing phenolic- and vitamin-loaded nanoemulsions for antiaging formulations,^206^ and scaling up bioactive delivery systems with superior reproducibility.^207^

Looking ahead, future research should prioritize hybrid AI models combining ANNs with deep learning architectures (e.g., convolutional neural networks) for high-throughput in silico screening of thousands of surfactant–bioactive combinations, enabling personalized and precision cosmetics. Real-time integration of ANNs with Industry 4.0 sensors will support adaptive manufacturing and Quality-by-Design (QbD) implementation, reducing waste and ensuring batch-to-batch consistency. Additionally, ANN-driven predictive toxicology will facilitate early assessment of long-term skin safety, allergenicity, and regulatory compliance, accelerating the translation of nanoemulsion prototypes into market-ready, sustainable, and high-performance cosmeceutical products.

### 8.1 Future Research Needs to Overcome Current Limitations

Despite these advances, several challenges remain. One key issue is ensuring the long-term safety and nontoxicity of nanoemulsions, particularly in products that are used daily on sensitive skin. Future research should focus on investigating the interaction between nanoemulsion-based cosmetics and the skin’s microbiome, as well as understanding the potential for bioaccumulation of nanoparticles. Additionally, there is a need for further studies to optimize the release kinetics and bioactivity of encapsulated compounds, as well as their stability under varying environmental conditions.^129^ Addressing these challenges will be crucial to unlocking the full potential of nanoemulsions in cosmetic products.

## Conclusions

This review has provided detailed information about the primary features of nanoemulsions referring to their formation, benefits as well as outstanding problems, and characterization techniques. Nanoemulsions have been widely studied in the cosmetic industry since they have potential outcomes in providing active ingredients in the skin safely and efficiently. Some of these properties include; small droplet size, larger surface area and increased stability which make it easy to enhance penetration through skin, controlled and specific release and delivery of active agents.

It is imperative, nevertheless, to observe that several factors can affect the nanoemulsions’ steadiness and efficiency. These aspects include; choice of surfactants, oils and cosurfactants, method of preparation including high-energy emulsification, microfluidization, and temperature and exposure to light. It therefore becomes relevant to grasp the effects of these factors to formulate and apply nanoemulsions in the best way possible.

Also, the effects of methods that are responsible for characterization of nanoemulsions should also be an area that requires extreme concern as far as the standard ways of analyzing its quality and stability are concerned. Nondestructive analytical methods, including dynamic light scattering, zeta potential analysis, and transmission electron microscopy, help toward characterizing the nanoemulsions techniques with regard to particle size, surface charge and morphology. Thus, the methods for characterization of nanoemulsions are developed and described in order to define the most important physicochemical characteristics of the materials and maintain the compliance and reproducibility of their properties.

Reflecting on these issues and progressing the development of nanoemulsion science, one can expand knowledge about nanoemulsions and apply them in cosmetics. Innovative features of nanoemulsions include formulations that create new possibilities in the skin care and beauty sector from skin conditions and remedy, improving skin moisturizing, all the way to skin protection from environmental factors. The authors explain the future developments in research and utilization of nanoemulsions applied to cosmetic products, promising to master more great breakthroughs in the way cosmetics activity work toward the dream of a healthy, appealing skin.

## Data Availability

All data generated or analyzed during this study are included in this published article.
